# Unraveling genetic sensitivity of beef cattle to environmental variation under tropical conditions

**DOI:** 10.1186/s12711-019-0470-x

**Published:** 2019-06-20

**Authors:** Roberto Carvalheiro, Roy Costilla, Haroldo H. R. Neves, Lucia G. Albuquerque, Stephen Moore, Ben J. Hayes

**Affiliations:** 1 School of Agricultural and Veterinarian Sciences, Sao Paulo State University (UNESP), Jaboticabal, SP 14884-900 Brazil; 2 National Council for Scientific and Technological Development (CNPq), Brasília, DF 71605-001 Brazil; 3 Institute for Molecular Bioscience (IMB), University of Queensland, St. Lucia, QLD 4072 Australia; 4 Queensland Alliance for Agriculture and Food Innovation (QAAFI), Centre for Animal Science, University of Queensland, St. Lucia, QLD 4072 Australia; 5 GenSys Associated Consultants, Porto Alegre, RS 90680-000 Brazil

## Abstract

**Background:**

Selection of cattle that are less sensitive to environmental variation in unfavorable environments and more adapted to harsh conditions is of primary importance for tropical beef cattle production systems. Understanding the genetic background of sensitivity to environmental variation is necessary for developing strategies and tools to increase efficiency and sustainability of beef production. We evaluated the degree of sensitivity of beef cattle performance to environmental variation, at the animal and molecular marker levels (412 K single nucleotide polymorphisms), by fitting and comparing the results of different reaction norm models (RNM), using a comprehensive dataset of Nellore cattle raised under diverse environmental conditions.

**Results:**

Heteroscedastic RNM (with different residual variances for environmental level) provided better fit than homoscedastic RNM. In addition, spline and quadratic RNM outperformed linear RNM, which suggests the existence of a nonlinear genetic component affecting the performance of Nellore cattle. This nonlinearity indicates that within-animal sensitivity depends on the environmental gradient (EG) level and that animals may present different patterns of sensitivity according to the range of environmental variations. The spline RNM showed that sensitivity to environmental variation from harsh to average EG is lowly correlated with sensitivity from average to good EG, at both the animal and molecular marker levels. Although the genomic regions that affect sensitivity in harsher environments were not the same as those associated with less challenging environments, the candidate genes within those regions participate in common biological processes such as those related to inflammatory and immune response. Some plausible candidate genes were identified.

**Conclusions:**

Sensitivity of tropical beef cattle to environmental variation is not continuous along the environmental gradient, which implies that animals that are less sensitive to harsher conditions are not necessarily less responsive to variations in better environmental conditions, and vice versa. The same pattern was observed at the molecular marker level, i.e. genomic regions and, consequently, candidate genes associated with sensitivity to harsh conditions were not the same as those associated with sensitivity to less challenging conditions.

**Electronic supplementary material:**

The online version of this article (10.1186/s12711-019-0470-x) contains supplementary material, which is available to authorized users.

## Background

Beef cattle production plays an important role in food security by converting forages and agricultural by-products into high-quality protein, and by optimizing land use through the occupation of grasslands that are unsuitable for agriculture [1, 2]. A large proportion of beef cattle are raised on tropical grasslands [3]. Under this production system, animals are usually subject to periods of food scarcity due to seasonal patterns of rainfall and, consequently, seasonal variation of pasture quality and availability. Depending on the severity of the dry season, the animals can be subject to seasonal weight loss if no supplementation is provided, this being a major constraint to beef cattle production in the tropics [4, 5]. However, supplementation is costly, and a more effective strategy would be to select cattle that are less sensitive to environmental variation in unfavorable environments and more adapted to harsh conditions.

Selection for reduced sensitivity to environmental variation can be addressed in different ways in animal breeding programs [6]. One alternative is to use reaction norm models (RNM), in which the response of each individual to environmental variation is modeled through a unique random regression curve that has its trajectory determined by a continuous environmental descriptor [7, 8]. For instance, RNM has been used to study heat tolerance in beef [9] and dairy cattle [10, 11] by modeling animals’ performance and the underlying breeding value as a function of a temperature-humidity index. In this application, animals with a smaller decline in production during high heat load were considered heat tolerant.

The choice of the type of curve to be used in RNM is of primary importance to properly assess the sensitivity of each animal to environmental variation. Generally, a linear regression is fitted since higher order polynomial coefficients may be difficult to estimate and interpret. However, if the change in phenotypic expression as a function of environmental variation is not constant along the environmental gradient, the random slope of a linear regression may not be a good indicator of the animal’s sensitivity. Therefore, testing higher order RNM is recommended [12].

Genotypic information has allowed extending RNM from the individual to the single nucleotide polymorphism (SNP) level [13–15]. Given a large dataset and a sufficient number of genotypes across the range of environments, the reaction norm of each SNP can reveal the size and direction of its effect on the trait of interest along the environmental gradient. An interesting feature of this approach would be to consider nonlinear reaction norms and investigate the possible existence of SNPs having environment-dependent sensitivity, i.e. SNPs that express more response to improvements in the environment and less response to its deterioration (and vice versa). This is particularly appealing in tropical beef production given the importance of genotype-by-environment interaction under these conditions [16].

Understanding the genetic background of sensitivity to environmental variation is critical in developing strategies to ameliorate current constraints on efficiency of beef cattle production in the tropics. This knowledge could be used, for example, in statistical models, which incorporate biological information to attain more accurate genomic predictions [17, 18]. The objective of our study was to unravel genetic sensitivity of beef cattle to environmental variation under tropical conditions. More specifically, we tested if sensitivity from harsh to average environment is similar to sensitivity from average to good environment, by comparing the fit of linear and non-linear RNM for post-weaning weight gain (PWG) of Nellore cattle. We tested this at the individual (animal) and molecular marker levels.

## Methods

### Phenotypes

Phenotypic and pedigree data were obtained from the Alliance Nellore dataset (www.gensys.com.br), which has information for over one million Nellore calves born between 1984 and 2016, from approximately 500 different commercial herds widely distributed in Brazil, Bolivia and Paraguay, which are highly related through the intensive adoption of artificial insemination (AI) (> 50% calves born from AI). This dataset is suitable to assess the sensitivity of beef cattle performance to environmental variation not only because of its size but also because of the diversity of management and environmental conditions in which the animals are raised. For instance, the average annual precipitation can vary from ~ 700 to ~ 3000 mm depending on the farm’s location, and the dry season in some regions may last up to 7 months. As mentioned previously, this variation of precipitation affects drastically the quality of the forage and, consequently, the animal’s performance since they are raised predominantly on pasture. Some farmers provide protein and mineral supplementation to the animals, especially during the dry season, but other farmers provide only urea to complement pasture.

We focused on the study of the post-weaning weight gain (PWG) trait because, after weaning, the animals are exposed to a wide range of environmental conditions, which makes PWG a suitable trait to assess sensitivity to environmental variation. For each animal, PWG was calculated as the difference between long-yearling and weaning weights, which were obtained at about 510 (310–730) and 210 (90–300) days of age, respectively. For the analysis, we considered only animals with known sires and dams, from contemporary groups (CG) with a minimum of 20 animals and PWG (adjusted for 300 days) between 30 and 250 kg. After filtering, the phenotypic dataset had individual PWG records for 421,585 animals from 9934 CG. Contemporary groups were formed by animals born in the same herd, year and season, from the same sex and raised in the same management group. The phenotyped animals were progeny of 6637 sires and 271,408 dams. The overall mean (sd) of PWG was 97.37 (33.13) kg. The distribution and other descriptive statistics of PWG are in Table S1 (see Additional file 1: Table S1) and in Figures S1 and S2 (see Additional file 2: Figures S1 and Additional file 3: Figure S2).

### Genotypes

Genotyping information for 13,806 Nellore cattle (*Bos indicus*) were used in genome-wide association (GWA) analyses. This information came from 1993 bulls, 4571 cows and 7242 progeny, which were genotyped with the Illumina BovineHD (HD) chip (~ 778 K SNPs; 4073 samples) or with a lower density (LD) chip (from ~ 28 to ~ 74 K SNPs; 9733 samples). A detailed list of the number of samples per chip is provided in Table S2 (see Additional file 1: Table S2). Lower density genotypes were imputed to HD using the software FImpute v.2.2 [19], with an expected imputation accuracy higher than 0.97 [20]. Only autosomal SNPs with a GenCall score, call rate and minor allele frequency higher than 0.15, 0.98 and 0.02, respectively, were used in the GWA analyses. We also discarded SNPs with a *p* value for Hardy–Weinberg equilibrium test lower than 10^−5^, and showing linkage disequilibrium (r^2^) greater than 0.998 with syntenic SNPs located within a window of 50 neighboring markers. After filtering, the total number of SNPs was equal to 412,456. All samples had a call rate higher than 0.90 (before imputation) and were kept for the GWA analyses.

### Environmental descriptor

Genetic sensitivity to environmental variation was assessed by reaction norm models (RNM) [7]. In this framework, the response of each animal to environmental variation is modeled as a unique curve that has its trajectory determined by a continuous environmental descriptor. In the absence of environmental descriptor information (e.g. temperature-humidity index or quality of pasture), descriptors derived from phenotypic data are commonly used (e.g. [21]). We opted for the use of the best linear unbiased estimates (BLUE) of CG effects as our environmental descriptor, since it encompasses the different management and environmental conditions to which the animals were exposed. In particular, we obtained BLUE from a regular mixed animal model [22] using PWG as the response variable, post-weaning age (linear and quadratic effects) and CG as fixed effects, and animal and residual as random effects. Estimated CG effects ranged from 31.38 to 225.16 kg, highlighting the diversity of conditions in which the animals were raised (~ sevenfold difference between extremes). The mean (sd) was 93.71 (28.16) kg. More information about CG solutions is in Figures S3 and S4 (see Additional file 4: Figures S3 and Additional file 5: Figure S4).

The environmental descriptor used on RNM, hereafter referred to as environmental gradient (EG), was the BLUE of CG effects that were standardized to have a zero mean and standard deviation (sd) equal to 1. Few CG (n = 72) had EG greater than 3 sd. Post-weaning gain records from those CG (n = 2728) were discarded for the subsequent analyses to avoid prejudicing the reaction norm estimates, since outliers can dramatically change the magnitude of regression coefficients [23]. After filtering, the average, minimum and maximum values of EG were equal to − 2.21, 0.0 and 3.0 sd, corresponding to CG effects on PWG equal to 31.5, 93.7 and 178.2 kg or, equivalently, to average daily gains (ADG) during the post-weaning period (300 days) equal to 105, 312 and 594 g/day, respectively. To attain this ADG over the full period, the animals most probably lost weight during the dry season (~ ½ post-weaning period) on lower EG, maintained their weight during the dry season on average EG, and gained weight even in the dry season on higher EG conditions. Thus, we will refer to lower, average and higher EG as harsh, average and good environments, respectively. However, it is important to keep in mind that the average EG consists of a challenging condition during the dry season. Progeny of genotyped sires comprised 78% of phenotypic records and, in general, they had records well distributed (within sire) across the range of EG (see Additional file 6: Figure S5). This is essential to obtain good reaction norm estimates at the marker level [24].

### Reaction norm models (RNM)

Five RNM were tested. The first applied RNM (RNM_homo) assumed that the residuals for the EG were homogenous and can be described by the equation:

$$y_{ij} = {\mathbf{x}}_{j}^{{\prime }} {\varvec{\upbeta}} + {\emptyset }_{1} \hat{w}_{i} + b_{{0_{j} }} + b_{{1_{j} }} \hat{w}_{i} + e_{ij} ,$$

where $y_{ij}$ is the phenotypic record (PWG) of animal *j* recorded in the level *i* of EG; ${\varvec{\upbeta}}$ is the vector of fixed effects (post-weaning period and CG), and ${\mathbf{x}} '_{j}$ is its corresponding row covariable/incidence vector; $\emptyset_{1}$ is the overall linear fixed regression coefficient of $y_{ij}$ on $\hat{w}_{i}$; $\hat{w}_{i}$ is the covariate associated to the *i*-th EG (estimated in the previous step); $b_{{0_{j} }}$ is the random overall additive genetic effect or the intercept of animal *j* for an average EG ($\hat{w} = 0$); $b_{{1_{j} }}$ is the random additive genetic effect of the reaction norm slope of animal *j* on $\hat{w}_{i}$ expressed as a deviation from $\emptyset_{1}$; and $e_{ij}$ is a random residual. RNM_homo was fitted under the assumptions:

$$\begin{aligned} & \left\{ {b_{{0_{j} }} ,b_{{1_{j} }} } \right\}\sim\,N(0,{\mathbf{A}} \otimes \left[ {\begin{array}{*{20}l} {\sigma_{{b_{0} }}^{2} } \hfill & {\sigma_{{b_{0} ,b_{1} }} } \hfill \\ {\sigma_{{b_{0} ,b_{1} }} } \hfill & {\sigma_{{b_{1} }}^{2} } \hfill \\ \end{array} } \right], \\ & {\text{and}}\;\left\{ {e_{ij} } \right\}\sim\,N\left( {0, {\mathbf{I}}\sigma_{e}^{2} } \right), \\ \end{aligned}$$

where ${\mathbf{A}}$ is the relationship matrix based on pedigree information ($\otimes$ is the Kronecker product); $\sigma_{{b_{0} }}^{2}$, $\sigma_{{b_{1} }}^{2}$ and $\sigma_{{b_{0} ,b_{1} }}$ are the variances of the intercept, the slope and their covariance, respectively; **I** is an identity matrix and $\sigma_{e}^{2}$ is the residual variance.

The second RNM (RNM_hete) was similar to RNM_homo except that the residuals were modeled assuming a specific residual variance for each EG, using a linear regression on $\hat{w}_{i}$. The heterogeneous residual variance coefficients for the intercept and slope were modelled using a log-residual function [25]. The third RNM (RNM_quad) was similar to RNM_hete except that it considered a polynomial quadratic regression to model the fixed curve and the reaction norm of the random effects (additive genetic and residual) instead of a linear regression. RNM_quad can be described by the equation:

$$y_{ij} = {\mathbf{x}}_{j}^{{\prime }} {\varvec{\upbeta}} + \emptyset_{1} \hat{w}_{i} + \emptyset_{2} \hat{w}_{i}^{2} + b_{{0_{j} }} + b_{{1_{j} }} \hat{w}_{i} + b_{{2_{j} }} \hat{w}_{i}^{2} + e_{ij},$$

where, $\emptyset_{2}$ is the overall quadratic fixed regression coefficient of $y_{ij}$ on $\hat{w}_{i}$; $\hat{w}_{i}^{2}$ is the squared value of $\hat{w}_{i}$; $b_{{2_{j} }}$ is the quadratic effect of the random additive genetic effect of the reaction norm of animal *j* on $\hat{w}_{i}$ expressed as a deviation from $\emptyset_{2}$; and all other terms as previously specified.

The other two tested RNM also fitted heterogeneous residual variances and used a linear–linear (RNM_l-l) and a quadratic–quadratic (RNM_q-q) spline function to model the fixed curve and the reaction norm of the additive genetic and residual random effects. For both spline functions we used only one knot (k) placed at the average EG (k = 0). RNM_l-l can be described by the equation:

$$y_{ij} = {\mathbf{x}}_{j}^{{\prime }} {\varvec{\upbeta}} + \emptyset_{1} \hat{w}_{i} + \emptyset_{1}^{*} \hat{w}_{i}^{*} + b_{{0_{j} }} + b_{{1_{j} }} \hat{w}_{i} + b_{{1_{j} }}^{*} \hat{w}_{i}^{*} + e_{ij},$$

where $\emptyset_{1}^{*}$ is the overall difference between the linear fixed regression coefficients of the first and second segments of the linear–linear spline function of $y_{ij}$ on $\hat{w}_{i}^{*}$ ($\hat{w}_{i}^{*} = 0$ if $\hat{w}_{i} < k$; $\hat{w}_{i}^{*} = \hat{w}_{i}$ if $\hat{w}_{i} \ge k$); $b_{{1_{j} }}^{*}$ is the difference between the reaction norm slopes of the first and second segments of the linear–linear spline function of the random additive genetic effect of animal *j* on $\hat{w}_{i}^{*}$ expressed as a deviation from $\emptyset_{1}^{*}$; and all other terms as previously specified. With this model parameterization, the estimates of the slope of the second segment ($b_{{1seg2_{j} }}$) were obtained as: $b_{{1seg2_{j} }} = b_{1j} + b_{{1_{j} }}^{*}$. RNM_q-q can be described by the equation:

$$\begin{aligned} y_{ij} & = {\mathbf{x}}_{j}^{{\prime }} {\varvec{\upbeta}} + \emptyset_{1} \hat{w}_{i} + \emptyset_{2} \hat{w}_{i}^{2} + \emptyset_{1}^{ *} \hat{w}_{i}^{ *} + \emptyset_{2}^{ *} \hat{w}_{i}^{2 *} \\ & \quad + b_{{0_{j} }} + b_{{1_{j} }} \hat{w}_{i} + b_{{2_{j} }} \hat{w}_{i}^{2} + b_{{1_{j} }}^{ *} \hat{w}_{i}^{ *} + b_{{2_{j} }}^{ *} \hat{w}_{i}^{2 *} + e_{ij} , \\ \end{aligned}$$

where $\emptyset_{2}^{*}$ is the overall difference between the quadratic fixed regression coefficients of the first and second segments of the quadratic–quadratic spline function of $y_{ij}$ on $\hat{w}_{i}^{2*}\ (\hat{w}_{i}^{2*} = 0 {\text{ if }} \hat{w}_{i} < k; \; \hat{w}_{i}^{2*} = \hat{w}_{i}^{2*} {\text{ if }}\hat{w}_{i} \ge k);\;b_{{2_{j} }}^{*}$ is the difference between the reaction norm quadratic effects of the first and second segments of the quadratic–quadratic spline function of the random additive genetic effect of animal *j* on $\hat{w}_{i}^{*}$ expressed as a deviation from $\emptyset_{2}^{*}$; and all other terms as previously specified. With this model parameterization, the quadratic effect estimates of the second segment ($b_{{2seg2_{j} }}$) were obtained as: $b_{{2seg2_{j} }} = b_{{2_{j} }} + b_{{2_{j} }}^{*}$.

Quadratic RNM (RNM_quad and RNM_q-q) were used to evaluate any advantage compared with linear reaction norms. Spline RNM (RNM_l-l and RNM_q-q) were used to evaluate if sensitivity in the first segment (harsh to average EG) is correlated with sensitivity in the second segment (average to good EG). Higher order polynomials were tested but convergence was not achieved (results not shown).

Estimates of (co)variance components of all RNM were obtained by restricted maximum likelihood using the AIREMLF90 software [26]. The different RNM were compared based on Akaike (AIC) [27] and Bayesian (BIC) [28] information criteria.

### Genome-wide association (GWA) analyses

GWA analyses were performed using the weighted single-step genomic BLUP method [29], which first predicts genomic estimated breeding values (GEBV) and then back-solve GEBV to SNP effects using equivalent models [30]. Two analyses were run using models similar to RNM_hete and RNM_l–l, but this time assuming that (co)variance components are known by using the corresponding estimates obtained in the previous step, and also replacing ${\mathbf{A}}$ by ${\mathbf{H}}$, a matrix which combines pedigree (${\mathbf{A}}$) and genomic (${\mathbf{G}}$) relationships [31]. Matrix ${\mathbf{G}}$ was computed as the first method proposed by [32]. Association analyses were not run for RNM_quad and RNM_q-q because SNP effects of the quadratic coefficient are difficult to interpret and also because sensitivity assessed as the slopes of RNM_l-l was highly correlated (0.97-0.99) with sensitivity measured as the first derivatives of RNM_quad and RNM_q-q. Association analysis was not run also for RNM_homo because it was the worst fitting model (as will be shown later).

For each GWA model (RNM_hete and RNM_l-l), SNP effects were iteratively recomputed weighting them proportionally to the genetic variance they explained in the previous iteration, resulting in an increased shrinkage of the SNPs explaining a lower variance and more pronounced effects of the SNPs explaining a higher proportion of genetic variance, compared to the previous iteration. Following [29], the same weight (equal to 1) was given to all SNPs in the first iteration and two additional iterations were performed. In addition to SNP effect estimates, the percentage of genetic variance explained by the segment of five adjacent SNPs was also computed for each SNP. GWA analyses were performed using BLUPF90 family programs [26].

### Functional enrichment analyses

Enrichment analyses were performed for the slopes of models RNM_hete (b1) and RNM_l-l (b1.seg1 and b1.seg2). For each slope, SNPs that explained at least 0.5% of the genetic variance were identified and genes that were located within 200 kb were annotated using Ensembl BioMart (Ensembl 93) [33] and UMD3.1 bovine reference genome [34]. Since a high proportion of genes within significant regions had human orthologs, functional enrichment analyses were performed with the GENE2FUNC process of the integrative web-based platform FUMA v1.3.3 [35], using all the human genes with bovine orthologs as background, totaling 18,425 genes with unique Entrez ID retrieved from the Ensembl genes 93 database. FUMA overrepresentation analyses were performed using hypergeometric tests and Benjamini–Hochberg multiple testing correction. Biological functions of genes with an adjusted enrichment *p* value ≤ 0.05 were reported. GENE2FUNC also provided enrichment of tissue-specific gene expression based on GTEx v6 RNA-seq data [36] using Bonferroni multiple test correction.

## Results

### Reaction norm models

Additive genetic variability was observed for the slope of RNM, which indicated the presence of genotype-by-environment interaction (G × E) on PWG (Table 1). Evidence of G × E was also observed in a preliminary multi-trait analyses, assuming PWG from three categories of EG (EG < −1; −1 ≤ EG ≤ 1; EG > 1) as different traits. For instance, the genetic correlation between PWG from harsh (EG < −1) and good (EG > 1) environments was equal to 0.79, in the multi-trait analyses (results not shown).

**Table 1:** Estimates of variance (block-diagonals), covariance (upper triangular blocks; in italic) and correlation (lower triangular blocks; in bold) among coefficients of reaction norm models (RNM) for the additive genetic effect of post-weaning weight gain (kg) in Nellore cattle, along with residual variance estimates and Akaike (AIC) and Bayesian (BIC) information criteria

Model^a^	Coefficient^b^	b0	b1	b2	b3	b4	e^c^	np^d^	AIC^e^	BIC^5^
RNM_homo	b0 (int)	129.73	*48.64*				223.18	4	3,560,790	3,560,838
	b1 (slp)	**0.96**	19.79							
RNM_hete	b0 (int)	89.80	*16.33*				5.55	5	3,559,272 (− 1518)	3,559,332 (− 1506)
	b1 (slp)	**0.86**	3.99				0.23			
RNM_quad	b0 (int)	95.90	*11.53*	− *6.75*			5.58	9	3,558,581 (− 2209)	3,558,689 (− 2149)
	b1 (slp)	**0.40**	8.72	*2.13*			0.24			
	b2 (qdr)	− **0.50**	**0.52**	1.92			− 0.06			
RNM_l-l	b0 (int)	102.86	*24.97*	− *1.00*			5.60	9	3,558,765 (− 2025)	3,558,873 (− 1965)
	b1 (slp1)	**0.89**	7.69	*0.71*			0.32			
	b2 (slp2)	− **0.02**	**0.06**	16.71			0.16			
RNM_q-q	b0 (int)	98.03	*24.51*	− *3.27*	− *1.55*	*0.82*	5.55	20	3,558,372 (− 2418)	3,558,611 (− 2227)
	b1 (slp1)	**0.31**	64.15	*25.80*	− *33.82*	*17.93*	0.06			
	b2 (qdr1)	− **0.09**	**0.91**	12.50	− *14.09*	*7.59*	− 0.18			
	b3 (slp2)	− **0.02**	− **0.48**	− **0.45**	78.93	− *28.23*	0.20			
	b4 (qdr2)	**0.02**	**0.66**	**0.63**	− **0.93**	11.63	− 0.01			

^a^RNM_homo: linear homoscedastic; RNM_hete: linear heteroscedastic; RNM_quad: quadratic heteroscedastic; RNM_l-l: spline linear–linear heteroscedastic; RNM_q-q: spline quadratic–quadratic heteroscedastic

^b^b0–b4 coefficients of the RNM for the additive genetic random effect [int: intercept; slp: slope; qdr: quadratic; slp1(2): slope segment 1(2); qdr1(2): quadratic segment 1(2)]

^c^Residual variance (RNM_homo) or residual coefficients associated with parameters of heteroscedastic RNM that were modeled using a log-residual function [25]

^d^Number of estimated parameters

^e^Numbers in parenthesis refer to difference in comparison with RNM_homo

Based on AIC and BIC criteria, all heteroscedastic RNM outperformed the homoscedastic model (Table 1). Among the heteroscedastic models, the quadratic (RNM_quad) and the spline models (RNM_l-l and RNM_q-q) outperformed the linear model (RNM_hete), which suggests the existence of a nonlinear component in the reaction norm of PWG in Nellore cattle. Model RNM_q-q had the best fit among the tested models according to both criteria, AIC and BIC.

Models RNM_homo and RNM_hete showed a high correlation (0.96 and 0.86, respectively) between intercept and slope (Table 1), which indicates that animals with better EBV for the average environment tended also to present higher sensitivity to environmental variation. However, correlation estimates showed by RNM_l-l suggest that this association is strong only for the first segment (harsh to average EG). In fact, the correlation was close to 0 for RNM_l-l between intercept and slope of the second segment (− 0.02) and between slopes of the first and second segments (0.06), whereas the correlation between intercept and slope of the first segment was 0.89 (Table 1).

**Fig. 1:**
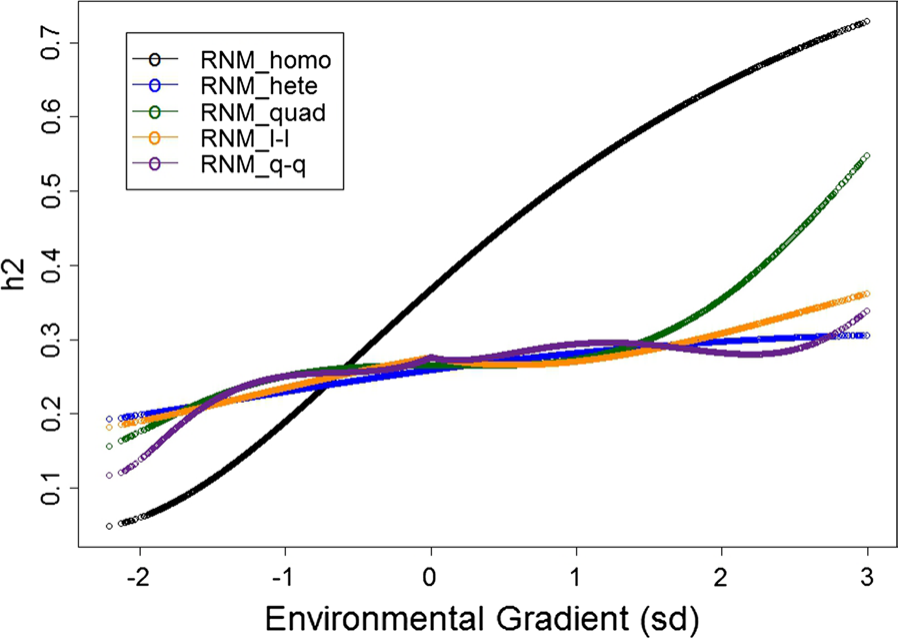
Heritability estimates (h2) for post-weaning weight gain of Nellore cattle according to the environmental gradient, for different reaction norm models. RNM_homo: linear homoscedastic; RNM_hete: linear heteroscedastic; RNM_quad: quadratic heteroscedastic; RNM_l-l: spline linear–linear heteroscedastic; RNM_q-q: spline quadratic–quadratic heteroscedastic

Increasing additive genetic variance (see Additional file 7: Figure S6) and heritability (Fig. 1) estimates from lower to higher EG were obtained for all models. Because homoscedastic residuals were assumed, RNM_homo overestimated heritability in good environments and underestimated it in harsh environments, compared to heteroscedastic models. All heteroscedastic models showed similar heritability estimates for intermediate EG (− 1.5 < EG < 1.5). Model RNM_quad presented higher heritability estimates for EG ≥ 2 and RNM_q-q showed lower heritability estimates for EG ≤ −2, in comparison with the other heteroscedastic models (Fig. 1).

**Fig. 2:**
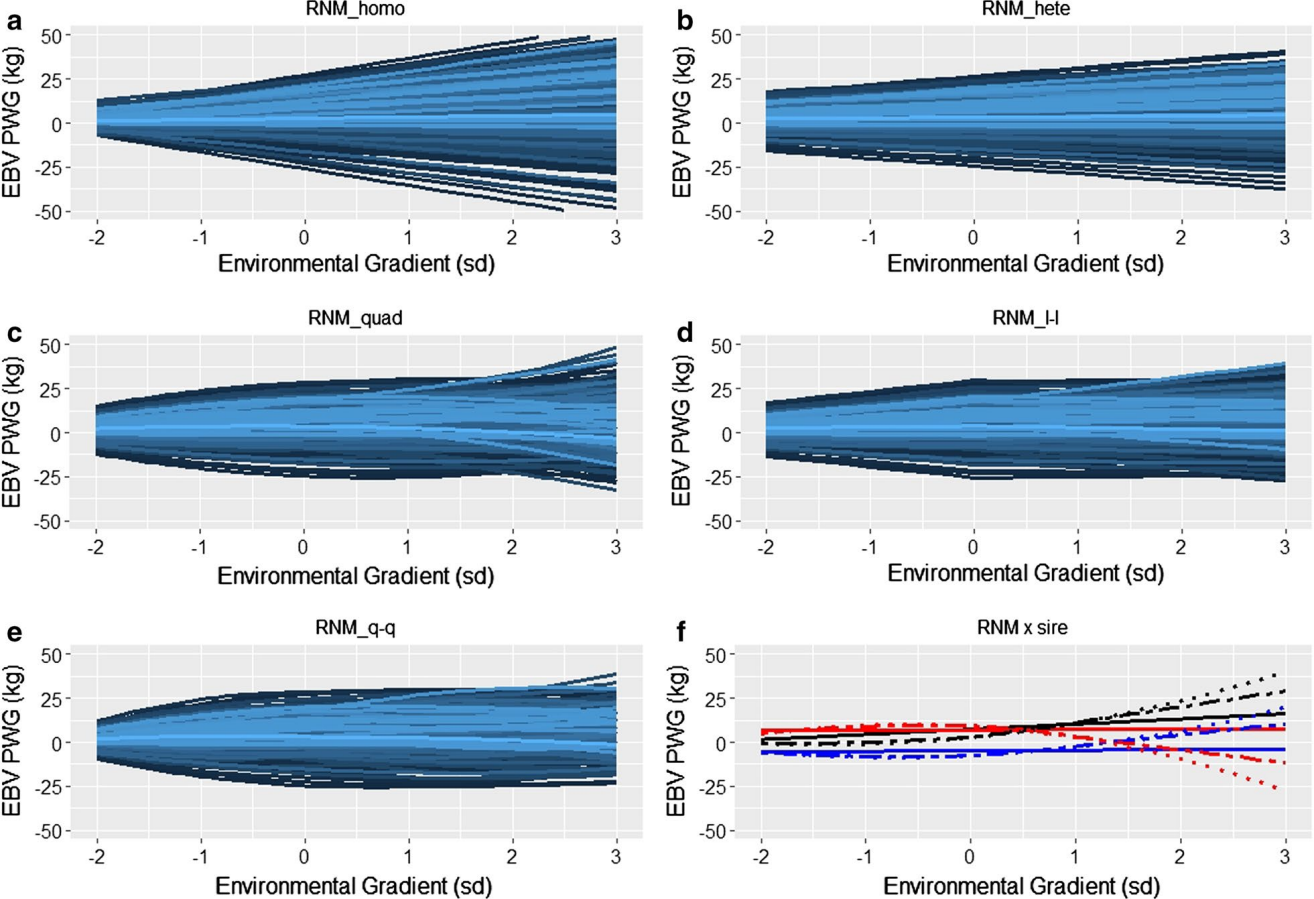
Estimated breeding values (EBV) for post-weaning weight gain (PWG) of Nellore cattle according to the environmental gradient, for different reaction norm models. RNM_homo: linear homoscedastic; RNM_hete: linear heteroscedastic; RNM_quad: quadratic heteroscedastic; RNM_l-l: spline linear–linear heteroscedastic; RNM_q-q: spline quadratic–quadratic heteroscedastic. **a**–**e** Reaction norms of genotyped sires with at least 50 progeny (n = 627). **f** Reaction norms of three selected sires (differentiated by color) for models RNM_hete (solid line), RNM_quad (dotted curve) and RNM_l-l (dashed curve)

Heritability estimates of RNM_homo resulted in over-shrinkage of EBV in lower EG, the opposite occurring with EBV in higher EG, compared to EBV of heteroscedastic models (Fig. 2). In accordance with (co)variance component estimates and model comparison results, there was further evidence from the comparison of EBV from the RNM_quad, RNM_l-l and RNM_q-q models that the nonlinear component in the reaction norm of PWG in Nellore cattle was important (Fig. 2c–e). The EBV from RNM_l-l illustrate that sensitivity to environmental variation from harsh to average EG tends to not be genetically correlated with sensitivity from average to good EG, in agreement with the genetic correlation estimates. Some animals were robust (i.e. had a flatter slope) for EG < 0 but were not robust for EG > 0, and vice versa (Fig. 2d). The same pattern was observed when PWG data from the two EG classes (EG < 0 and EG > 0) were analyzed independently, using two separate heteroscedastic linear regression RNM (see Additional file 8: Figure S7), which reinforces the plausibility of the RNM_l-l results. Figure 2f illustrates that the different models can provide quite different EBV rankings between animals, especially at higher EG levels.

### Genome-wide association and functional enrichment

**Fig. 3:**
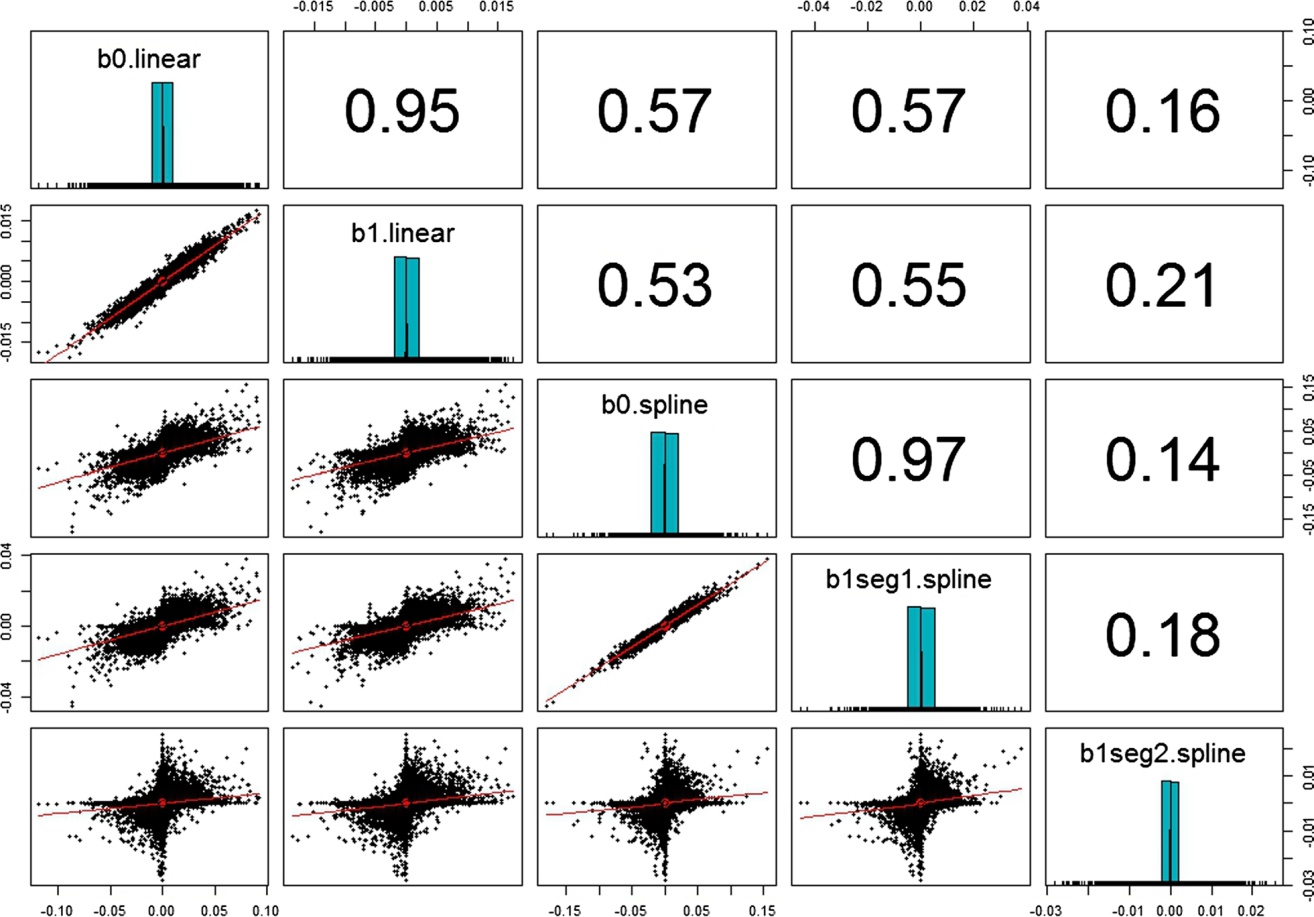
SNP effect estimates distribution (diagonal), correlation (upper triangular) and scatter plot (lower triangular) for coefficients of reaction norm models (RNM). b0.linear: intercept of RNM_hete; b1.linear: slope of RNM_hete; b0.spline: intercept of RNM_l-l; b1seg1(2).spline: slope segment 1(2) of RNM_l-l; RNM_hete: linear heteroscedastic RNM; RNM_l-l: spline linear–linear heteroscedastic RNM. X-axis and y-axis (lower triangular): SNP effect estimates (kg); y-axis (diagonal): frequency

Estimates of SNP effects on the intercept and slope of RNM_hete were highly correlated (0.95), suggesting that average performance and sensitivity of PWG would have a similar genetic background. However, estimates of SNP effects obtained with RNM_l-l indicate that this similarity holds only for sensitivity from harsh to average environmental variation (r = 0.97), whereas a low correlation (0.14) was observed between estimates of SNP effects of the intercept and slope of the second segment (Fig. 3).

Twenty-three genomic regions, from 16 chromosomes, were identified containing segment(s) of five adjacent SNPs that explained 0.5% or more of the genetic variance for at least one of the coefficients of RNM_hete and RNM_l-l (Table 2) (see Additional file 9: Figure S8). These regions, with a size ranging from 400.0 to 436.6 kb (200 kb upstream and downstream the significant SNP(s)), include 54 annotated genes in total. The numbers of regions (genes) associated with the intercept and slope of RNM_hete were equal to 7 (19) and 6 (13), respectively, and the numbers of regions (genes) associated with the intercept and the slopes of the first and second segments of RNM_l-l were equal to 7 (12), 7 (10) and 11 (30), respectively. In accordance with the results of correlation between effects of SNPs, there were overlapping candidate regions for the intercept and slope of RNM_hete (5) and for the intercept and slope of the first segment of RNM_l-l (6). No overlapping candidate regions were observed between slopes of RNM_l-l or between its intercept and slope of the second segment (Table 2).

**Table 2:** Genomic regions associated with coefficients of reaction norm models for post-weaning weight gain in Nellore cattle, and annotated genes within those regions

Region	%Var^a^					Chr^b^	Window start^b^ (bp)	Window end^b^ (bp)	Gene stable ID^c^	Gene symbol^c^
	b0	b1	b0s	b1s1	b1s2					
1			0.55	0.55		1	84,867,382	85,267,382	–	–
2					0.58	3	35,940,081	36,344,427	*ENSBTAG00000014530*	*NTNG1*
3					0.78	3	96,120,576	96,520,576	*ENSBTAG00000014482*	*FAF1*
									ENSBTAG00000045402	RF00322
									*ENSBTAG00000010414*	*DMRTA2*
4				0.55		3	114,849,177	115,249,177	*ENSBTAG00000013869*	*SH3BP4*
5	0.56		1.52	1.61		4	8,329,351	8,741,255	*ENSBTAG00000002107*	*FZD1*
									*ENSBTAG00000027319*	
6	1.00	0.88	1.25	1.14		5	84,442,697	84,878,158	ENSBTAG00000039906	
									ENSBTAG00000046108	
									ENSBTAG00000000749	LMNTD1
7					0.74	7	22,728,136	23,164,748	*ENSBTAG00000009034*	*AP3D1*
									*ENSBTAG00000025448*	*IZUMO4*
									*ENSBTAG00000014526*	*MOB3A*
									*ENSBTAG00000018049*	*MKNK2*
									ENSBTAG00000025445	
									*ENSBTAG00000008609*	*SEPT8*
									*ENSBTAG00000031246*	*CCNI2*
									*ENSBTAG00000025477*	*KIF3A*
									*ENSBTAG00000015957*	*IL4*
									*ENSBTAG00000015953*	*IL13*
									*ENSBTAG00000011252*	*RAD50*
8			0.76	0.75		7	95,572,442	95,972,442	–	–
9					0.82	8	107,325,318	107,747,332	*ENSBTAG00000004010*	*PAPPA*
									*ENSBTAG00000047511*	
									*ENSBTAG00000025667*	
									*ENSBTAG00000017155*	*TRIM32*
10					0.53	9	58,226,166	58,643,182	–	–
11					0.50	10	7,290,264	7,690,264	*ENSBTAG00000017507*	*SV2C*
									*ENSBTAG00000000897*	*IQGAP2*
12	0.79	0.63				11	22,788,685	23,203,999	*ENSBTAG00000013861*	*SLC8A1*
									ENSBTAG00000037153	RF00001
13	0.60	0.63	0.68	0.55		11	56,219,017	56,652,372	*ENSBTAG00000007135*	*REG3G*
									*ENSBTAG00000011314*	*REG3A*
14	0.56	0.64	0.77	0.74		14	19,482,365	19,901,806	ENSBTAG00000044730	RF00026
									*ENSBTAG00000019892*	*HAS2*
15					0.50	14	72,964,805	73,364,805	–	–
16					0.83	16	64,552,728	64,958,425	*ENSBTAG00000046243*	*ZNF648*
									ENSBTAG00000027426	
									*ENSBTAG00000013631*	*GLUL*
17		0.50				17	7,729,955	8,129,955	*ENSBTAG00000008098*	*DCLK2*
18			0.51			17	11,993,902	12,393,902	ENSBTAG00000034522	SLC10A7
									ENSBTAG00000048020	
									ENSBTAG00000014060	LSM6
19	0.54					18	59,112,039	59,512,039	ENSBTAG00000045571	
									ENSBTAG00000037440	
									ENSBTAG00000047712	
									ENSBTAG00000030454	
									ENSBTAG00000011052	
20	0.84	0.71				20	65,401,804	65,809,687	*ENSBTAG00000009401*	*MTRR*
									*ENSBTAG00000009400* *ENSBTAG00000019210*	*FASTKD3* *ADCY2*
21					0.63	27	11,730,389	12,130,389	*ENSBTAG00000047427*	*RF00100*
22					0.52	27	32,310,351	32,710,351	*ENSBTAG00000033381*	
									*ENSBTAG00000013943*	*ZNF703*
23					0.62	29	12,876,785	13,278,418	*ENSBTAG00000033731*	*PRCP*
									*ENSBTAG00000006812*	*FAM181B*
									ENSBTAG00000044832	

^a^Percentage of genetic variance (%Var) explained by the leading segment (within region) of five adjacent SNPs, for each coefficient. b0: intercept of a heteroscedastic linear reaction norm model (RNM_hete); b1: slope of RNM_hete; b0s: intercept of a heteroscedastic spline linear–linear reaction norm model (RNM_l-l); b1s1: slope of segment 1 of RNM_l-l; b1s2: slope of segment 2 of RNM_l-l. Only  %Var ≥ 0.5% are presented

^b^Chromosome (Chr) and bp position (bpp) according to the UMD3.1 assembly

^c^Retrieved from the BioMart Ensembl genes 93 database; genes with human orthologs, used for enrichment analyses, are presented in italics

From the 49 genes associated with the slopes of models RNM_hete or RNM_l-l, 37 (75.5%) have human orthologs (Table 2) and were used for functional enrichment analyses. Seven from the eight genes with human orthologs associated with the slope of RNM_hete (b1) had 48 significantly enriched biological processes (BP) (see Additional file 10: Figure S9). The BP associated with this set of genes (*ADCY2*, *DCLK2*, *HAS2*, *MTRR*, *REG3A*, *REG3G* and *SLC8A1*) were predominantly related to epithelial, inflammatory, homeostatic, and cell proliferation and differentiation processes. For the slope of the first segment of RNM_l-l (b1seg1), five genes (*HAS2*, *REG3A*, *REG3G*, *FZD1* and *SH3BP4*), out of six with human orthologs, showed 35 significantly enriched BP, predominantly related to epithelial, inflammatory, nitrogen compound, and cell proliferation and differentiation processes (see Additional file 11: Figure S10). For the slope of the second segment of RNM_l-l (b1seg2), 16 genes (*AP3D1*, *ASTN2*, *DMRTA2*, *FAF1*, *GLUL*, *IL4*, *IL13*, *IQGAP2*, *KIF3A*, *MKNK2*, *PAPPA*, *PRCP*, *RAD50*, *SV2C*, *TRIM32* and *ZNF703*), out of 26 with human orthologs, showed 104 significantly enriched BP (the majority associated with *IL4* and *IL13*), predominantly related to immune response, metabolic, and cell death, proliferation and differentiation processes (see Additional file 12: Figure S11).

GENE2FUNC gene expression results evidenced that the human orthologs of the bovine genes associated with sensitivity assessed by the slope of RNM_hete (b1) tend to be more differently expressed in humans in the small intestine and adrenal gland (see Additional file 13: Figure S12). For the genes associated with the slope of the first segment of RNM_l-l, GENE2FUNC identified significant enrichment of up-regulated genes in the human pancreas (see Additional file 14: Figure S13). For sensitivity assessed by the slope of the second segment of RNM_l-l, the set of candidate genes tended to be more differently expressed in humans in the brain (see Additional file 15: Figure S14).

## Discussion

### Reaction norm models

When running genetic evaluations, genetic sensitivity to environmental variation can be addressed in different ways in an animal breeding context. In this study, we used RNM to model the response to environmental variation of the trait PWG in Nellore cattle raised under tropical pasture conditions. The existence of additive genetic variability for the slope of RNM indicated the presence of G × E on PWG. It offers the possibility of using the individual random slopes as a selection criterion for robustness, since animals with a flatter slope are less sensitive to environmental variation. Other studies have also identified, through RNM, the importance of G × E for different traits in Nellore cattle [37–39].

An interesting modelling alternative to our environmental descriptor is a Bayesian hierarchical RNM (BHRNM). This model has been recommended when the environmental descriptor needs to be derived from phenotypic data [40, 41]. We chose not to use BHRNM because it would have drastically increased the computational time required for the analyses. Moreover, there is also evidence in the literature that “two-step” RNM (using BLUE previously estimated as environmental covariates) achieves a similar fit to BHRNM [42].

As in [37], we also observed that heteroscedastic RNM provided a better fit than homoscedastic RNM. In addition, we found that spline and quadratic RNM outperformed linear RNM, which suggests the existence of a nonlinear component in the reaction norm of PWG in Nellore cattle. This nonlinearity indicates that within-animal sensitivity depends on the EG level and that animals may present different patterns of sensitivity depending on the degree of EG. For instance, some animals may be less sensitive to variation in harsher environmental gradients and more sensitive to variation in not so challenging environmental gradients, and vice versa. This nonlinearity in the reaction norm seems to be less significant in cattle that are raised in more intensive systems, at least in dairy cattle [43], possibly because environmental factors that influence intensive systems tend to be more controlled, compared to pasture-based systems, resulting in a smaller environmental variation.

The high genetic correlation estimate between the intercept and slope of RNM_hete suggested little opportunity of joint selection for increased performance and reduced sensitivity. A similar result was also observed by [41], in a study on PWG of Angus cattle raised in Brazil. However, the results of RNM_l-l revealed that this high correlation holds only for harsher environments and that there are good opportunities to select for increased PWG and reduced sensitivity in not so challenging environmental conditions.

An alternative to select for increased PWG and reduced sensitivity jointly is to identify and select the best genotype for specific environmental conditions. However, this strategy may provide suboptimal genetic gain especially for tropical pasture systems where environmental conditions can vary substantially among years, even within farms. This reinforces the relevance of using sensitivity as a selection criterion.

### Genome-wide association and functional enrichment

There is strong evidence that the mechanisms associated with sensitivity depend on the degree of EG. GWAS results of RNM_l-l revealed that important genomic regions associated with sensitivity from harsh to average environmental conditions differed from those associated with sensitivity from average to good environmental conditions. This result is not surprising given the diversity of environmental conditions in which the animals were raised and the complexity of livestock response to stressful conditions [44].

In harsher environments, genes associated with acute inflammatory response and keratinocyte proliferation and differentiation processes seem to play an important role in sensitivity of beef cattle that is assessed as the slope of the first segment of RNM_l-l (b1seg1). For instance, the *regenerating islet*-*derived 3 alpha* (*REG3A*) gene is associated (in humans and mice) with wound repair after skin injury and with homeostasis of the skin, thus contributing to immune defense [45]. Another candidate gene of the REG3 family (*REG3G*), also associated with sensitivity to harsher environments (b1seg1), was found to be related with antimicrobial defense of the mammalian intestine [46] and with intestinal strategies for maintaining symbiotic host-microbial relationships [47]. The barrier function of gastrointestinal epithelium in host protection is well documented in the literature [48, 49] and severe feed restriction has been shown to compromise the total tract barrier function in beef cattle [50]. These results reinforce the plausibility of *REG3A* and *REG3G* being associated with sensitivity of beef cattle to harsh environments. Another candidate gene potentially associated with sensitivity to harsh environmental conditions is the *SH3 domain binding protein 4* (*SH3BP4*). This gene was identified in a region with copy number variation in South African Nguni cattle, which are recognized for their ability to sustain harsh environmental conditions and resistance to parasites and disease [51].

In not so harsh environments, candidate genes associated with inflammatory and immune response seem also to play an important role in sensitivity of beef cattle that is assessed as the slope of the second segment of RNM_l-l (b1seg2). Among them, the *interleukin 4* (*IL4*) and *interleukin 13* (*IL13*) are perhaps the most plausible candidates. Both genes are known to share a wide range of activities on monocytes, epithelial cells and B cells, and thus play an important role in host defense [52–55]. Although interleukins and other cytokines are mainly reported to be involved in the regulation of immune response, there is also evidence about their role in regulating protein metabolism and muscle function [56], among other metabolic functions as, for example, energy homeostasis [57, 58].

Members of the heat shock protein (HSP) family have been considered as candidate genes for sensitivity or stress response in different studies [59–62]. No HSP genes were observed in our candidate regions. However, for sensitivity in not so harsh environments (b1seg2), the *glutamate*-*ammonia ligase* (*GLUL*) was identified as a candidate gene. *GLUL* is involved in glutamine synthesis, which, in turn, has been shown to be a powerful effector of HSP expression [63]. *GLUL* has also been reported to be associated with cellular response to starvation [64].

Other important candidate genes associated with b1seg2 are the: *adaptor related protein complex 3 subunit delta 1* (*AP3D1*), *kinesin family member 3A* (*KIF3A*) and *RAD50 double strand break repair protein* (*RAD50*). *AP3D1* is involved in the regulation of the sequestering of zinc ion, and zinc has been shown to have multiple impacts on the immune function [65]. *KIF3A* is upregulated in heat-shocked Holstein oocytes [66] and differently expressed in a bovine tick-resistance study [67]. *RAD50* is a key gene for the immune system [68] and was identified as candidate gene for harsh environmental adaptation in cattle [69].

Eleven out of the 30 genes associated with sensitivity to not so harsh environments (b1seg2) were from the same region between 22.7 and 23.2 Mb on chromosome 17. The genes within this region are highly conserved in different species and multiple organisms have orthologs with those genes (NCBI databases; www.ncbi.nlm.nih.gov). In humans, they are located in two regions, according to the reference assembly GRCh38.p12, one on chromosome 5 between 132.6 and 132.8 Mb (*SEPT8*, *CCNI2*, *KIF3A*, *IL4*, *IL13*, *RAD50*) and another on chromosome 19 between 2.0 and 2.2 Mb (*AP3D1*, *IZUMO4*, *MOB3A*, *MKNK2*). In Nellore cattle, the markers within this region are in high linkage disequilibrium (see Additional file 16: Figure S15). It is important to note that this candidate region would not have been identified, if we had performed GWAS using only RNM_hete.

Enrichment results from FUMA showed that besides the differences in candidate regions associated with b1seg1 and b1seg2, the genes in those regions also differ in their gene expression profile. This result cannot be extrapolated to cattle since it is based on human data, but can serve as a guide for future gene expression studies in cattle that aim at identifying differentially expressed genes associated with adaptation or sensitivity. Based on the results of the FUMA analysis, in harsher environments, sampling of gastrointestinal and pancreas tissues is recommended. In not so harsh but still challenging environments, sampling of brain and skin tissues is also recommended.

## Conclusions

Our results reveal important G × E interactions that affect the performance of beef cattle raised under tropical conditions or, more specifically, that G × E interactions affect PWG in Nellore cattle. Sensitivity to environmental variation at the individual level is not linear along the environmental gradient, since animals that are less sensitive to environmental changes under harsher conditions are not necessarily less responsive to not so challenging conditions, and vice versa. The same pattern was observed at the molecular marker level, i.e. genomic regions associated with sensitivity to harsh conditions are not the same as those associated with sensitivity to not so harsh (but still challenging) conditions, although the genes within those regions share common biological processes, such as those related with inflammatory and immune response. These results highlight the diversity and complexity of mechanisms that are involved in cattle response to stressful/unfavorable conditions, and should serve as a basis for future studies aiming at unraveling the genetic control of sensitivity.

## Additional files

**Additional file 1.** **Table S1** Descriptive statistics for post-weaning weight gain (kg), adjusted for 300 days, of Nellore cattle. **Table S2** Number of genotyped samples per SNP chip and animal category.

**Additional file 2: Figure S1.** Number of animals (counts) with own records for post-weaning weight gain, by year of birth and sex.

**Additional file 3: Figure S2.** Smoothed density plots of post-weaning weight gain (PWG), adjusted for a period of 300 days, of Nellore cattle.

**Additional file 4: Figure S3.** Smoothed density plots of contemporary group (CG) solutions (best linear unbiased estimates) for post-weaning weight gain (kg) of Nellore cattle.

**Additional file 5: Figure S4.** Boxplot of contemporary group (CG) solutions (best linear unbiased estimates) for post-weaning weight gain (kg) of Nellore cattle, by year of birth and sex.

**Additional file 6: Figure S5.** Scatterplot and histograms of progeny’s environmental gradient (EG) standard deviation (sd) and range (max(EG)-min(EG)), by genotyped sire with at least 5 progeny (n = 1384).

**Additional file 7: Figure S6.** Additive (left) and residual (right) variance component estimates for post-weaning weight gain of Nellore cattle according to the environmental gradient, for different reaction norm models. RNM_homo: linear homoscedastic; RNM_hete: linear heteroscedastic; RNM_quad: quadratic heteroscedastic; RNM_l-l: spline linear–linear heteroscedastic; RNM_q-q: spline quadratic–quadratic heteroscedastic.

**Additional file 8: Figure S7.** Estimated breeding values (EBV) for post-weaning weight gain (PWG) of Nellore cattle according to the environmental gradient (EG), obtained with a heteroscedastic linear reaction norm model (RNM_hete). To generate the results of this plot, data of the two segments (EG < 0 and EG > 0) were analyzed separately, running two independent RNM_hete analyses. The correlation estimates between the intercept (b0) and slope of the two segments (b1.seg1 and b1.seg2) are provided. Reaction norms of genotyped sires with at least 50 progeny and in common among the two EG segments (n = 621) are presented.

**Additional file 9: Figure S8.** Manhattan plots of percentage of genetic variance explained by the segment of 5 adjacent SNPs, for each parameter of the reaction norm models RNM_hete and RNM_l-l (RNM_hete: heteroscedastic linear; RNM_l-l: heteroscedastic spline linear–linear). The horizontal dashed line at 0.5 represents the empirical threshold used to identify candidate genomic regions (≥ 0.5).

**Additional file 10: Figure S9.** Significantly enriched gene ontology biological processes, identified by GENE2FUNC process of FUMA, associated with candidate genes for sensitivity to environmental variation of post-weaning weight gain in Nellore cattle, assessed by a random slope (b1) of a heteroscedastic linear random regression model (RNM_hete).

**Additional file 11: Figure S10.** Significantly enriched gene ontology biological processes, identified by GENE2FUNC process of FUMA, associated with candidate genes for sensitivity to environmental variation of post-weaning weight gain in Nellore cattle, assessed by a random slope of the first segment (b1seg1) of a heteroscedastic spline linear–linear random regression model (RNM_l-l).

**Additional file 12: Figure S11.** Significantly enriched gene ontology biological processes, identified by GENE2FUNC process of FUMA, associated with candidate genes for sensitivity to environmental variation of post-weaning weight gain in Nellore cattle, assessed by a random slope of the second segment (b1seg2) of a heteroscedastic spline linear–linear random regression model (RNM_l-l).

**Additional file 13: Figure S12.** Enrichment of human tissue specific gene expression based on GTEx v6 RNA-seq data [36], obtained with GENE2FUNC process of FUMA, for candidate ortholog bovine genes associated with sensitivity to environmental variation of post-weaning weight gain in Nellore cattle, assessed by a random slope (b1) of a heteroscedastic linear random regression model (RNM_hete).

**Additional file 14: Figure S13.** Enrichment of human tissue specific gene expression based on GTEx v6 RNA-seq data [36], obtained with GENE2FUNC process of FUMA, for candidate ortholog bovine genes associated with sensitivity to environmental variation of post-weaning weight gain in Nellore cattle, assessed by a random slope of the first segment (b1seg1) of a heteroscedastic spline linear–linear random regression model (RNM_l-l). Significant enrichment at Bonferroni corrected P ≤ 0.05 are colored in red.

**Additional file 15: Figure S14.** Enrichment of human tissue specific gene expression based on GTEx v6 RNA-seq data [36], obtained with GENE2FUNC process of FUMA, for candidate ortholog bovine genes associated with sensitivity to environmental variation of post-weaning weight gain in Nellore cattle, assessed by a random slope of the second segment (b1seg2) of a heteroscedastic spline linear–linear random regression model (RNM_l-l).

**Additional file 16: Figure S15.** Linkage disequilibrium (r^2^) between markers of the 22.7-23.2 Mb region of chromosome 7 (UMD 3.1 assembly), in Nellore cattle.

## Data Availability

All the data supporting the results of this study are included in the article and in the Additional files. The phenotypic and genotypic data cannot be shared because they are owned by commercial breeding programs.
