# Supplementary material for: Unraveling genetic sensitivity of beef cattle to environmental variation under tropical conditions
Source: Genet Sel Evol. 2019 Jun 20;51:29. doi: 10.1186/s12711-019-0470-x (PMC6585094; doi:10.1186/s12711-019-0470-x)
Supplement: Supplementary file 1 — Additional file 1. Table S1 Descriptive statistics for post-weaning weight gain (kg), adjusted for 300 days, of Nellore cattle. Table S2 Number of genotyped samples per SNP chip and animal category. [file 12711_2019_470_MOESM1_ESM.docx]

Table S1. Descriptive statistics for post-weaning weight gain (kg), adjusted for 300 days, of Nellore cattle.

| Sex | N | Mean | Standard deviation | Minimum | Maximum |
| --- | --- | --- | --- | --- | --- |
| Female | 212,323 | 86.58 | 28.02 | 30.00 | 249.93 |
| Male | 209,262 | 108.32 | 34.32 | 30.00 | 249.98 |
| All | 421,585 | 97.37 | 33.13 | 30.00 | 249.98 |

Table S2. Number of genotyped samples per SNP chip^1^ and animal category.

| Category | HD | GGPHDi | GGPLDi | 50K | Clarifide | Z-Chip | Total |
| --- | --- | --- | --- | --- | --- | --- | --- |
| Bull | 1008 | 374 | 105 | 426 | 26 | 54 | 1993 |
| Cow | 2531 | 759 | 30 | 604 | 273 | 374 | 4571 |
| Progeny | 534 | 1922 | 323 | 404 | 2461 | 1598 | 7242 |
| Total | 4073 | 3055 | 458 | 1434 | 2760 | 2026 | 13806 |

^1^HD: Illumina BovineHD (HD) (~778K SNPs); GGPHDi: GeneSeek Genomic Profiler (GGP) HD *Bos indicus* (~74K SNPs); GGPLDi: GGP LD *Bos indicus* (~30K SNPs); 50K: Illumina BovineSNP50 (~55K SNPs); Clarifide: Clarifide Nelore (~30K SNPs); Z-Chip: Deoxi LD

*Bos indicus* (~28K SNPs).
