# Supplementary figures and images for: Unraveling genetic sensitivity of beef cattle to environmental variation under tropical conditions
Source: Genet Sel Evol. 2019 Jun 20;51:29. doi: 10.1186/s12711-019-0470-x (PMC6585094; doi:10.1186/s12711-019-0470-x)

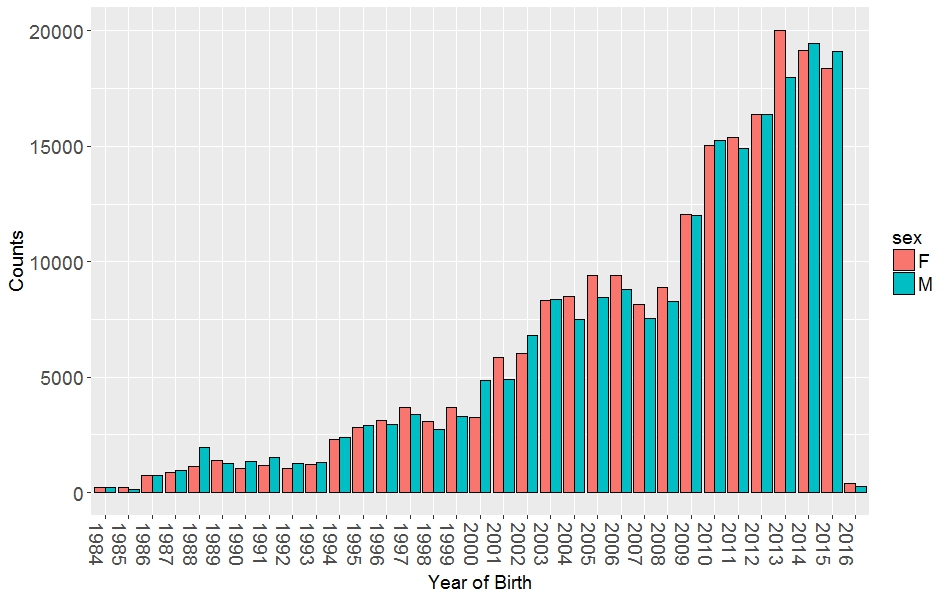

Supplement: Supplementary file 2 — Additional file 2: Figure S1. Number of animals (counts) with own records for post-weaning weight gain, by year of birth and sex. [file 12711_2019_470_MOESM2_ESM.jpeg]

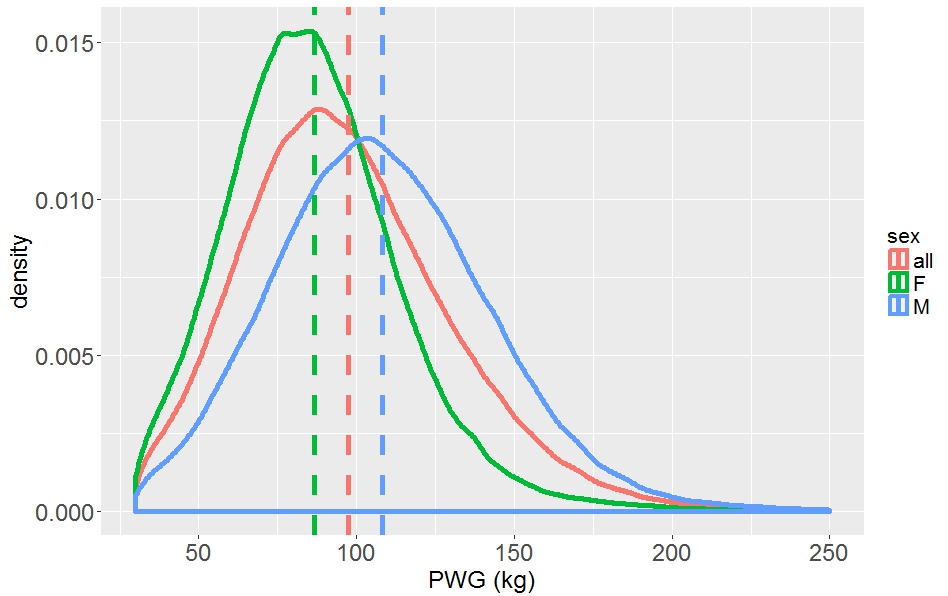

Supplement: Supplementary file 3 — Additional file 3: Figure S2. Smoothed density plots of post-weaning weight gain (PWG), adjusted for a period of 300 days, of Nellore cattle. [file 12711_2019_470_MOESM3_ESM.jpeg]

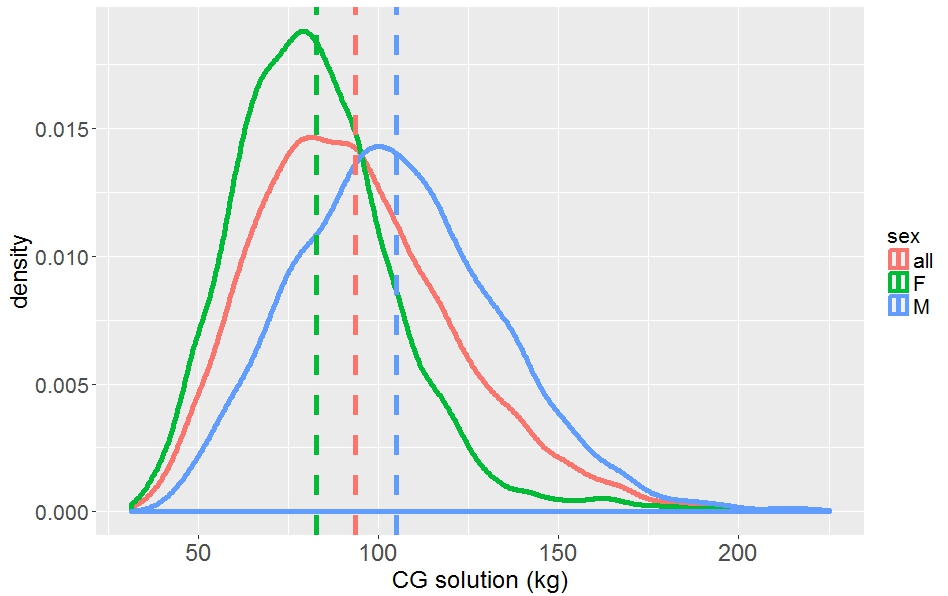

Supplement: Supplementary file 4 — Additional file 4: Figure S3. Smoothed density plots of contemporary group (CG) solutions (best linear unbiased estimates) for post-weaning weight gain (kg) of Nellore cattle. [file 12711_2019_470_MOESM4_ESM.jpeg]

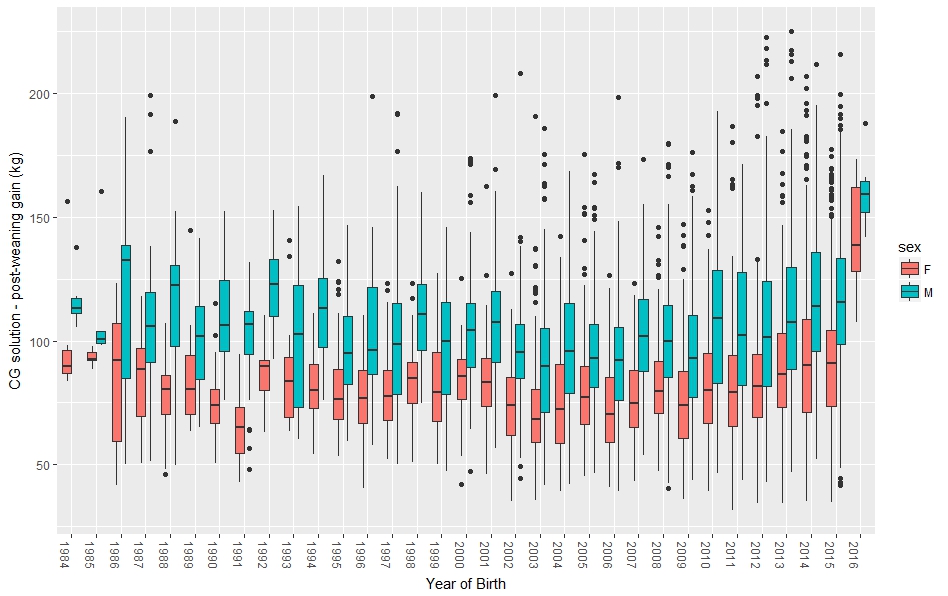

Supplement: Supplementary file 5 — Additional file 5: Figure S4. Boxplot of contemporary group (CG) solutions (best linear unbiased estimates) for post-weaning weight gain (kg) of Nellore cattle, by year of birth and sex. [file 12711_2019_470_MOESM5_ESM.jpeg]

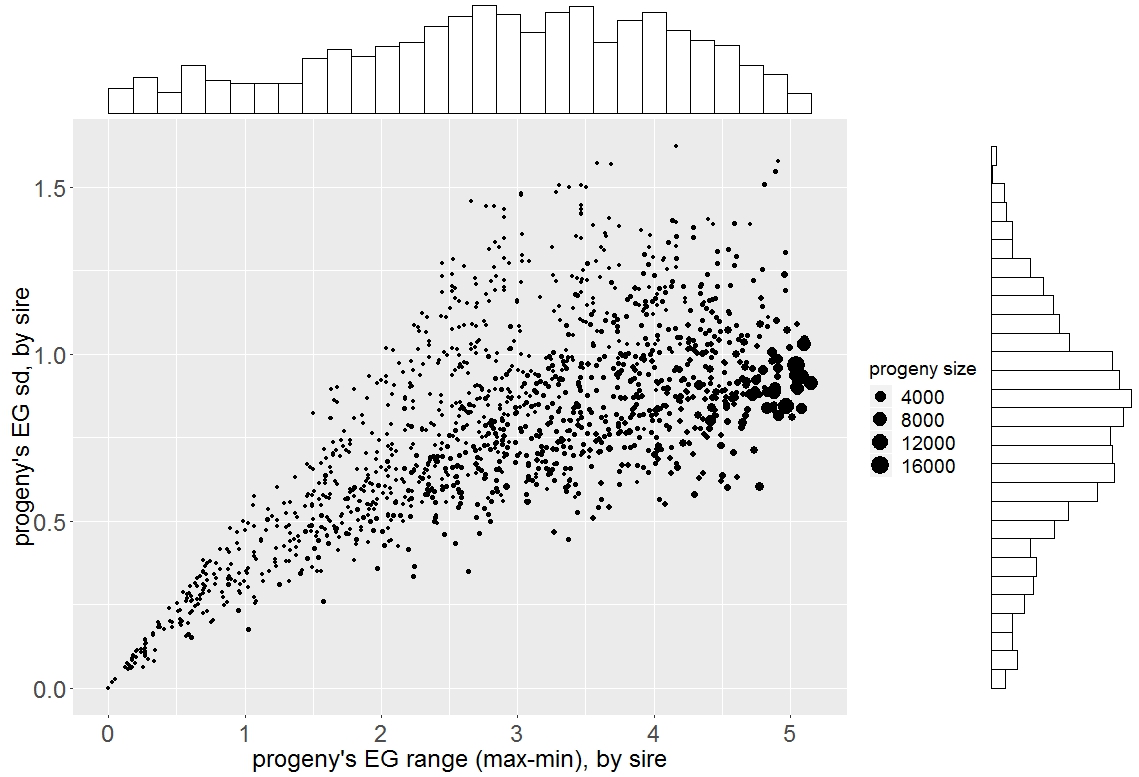

Supplement: Supplementary file 6 — Additional file 6: Figure S5. Scatterplot and histograms of progeny’s environmental gradient (EG) standard deviation (sd) and range (max(EG)-min(EG)), by genotyped sire with at least 5 progeny (n = 1384). [file 12711_2019_470_MOESM6_ESM.jpeg]

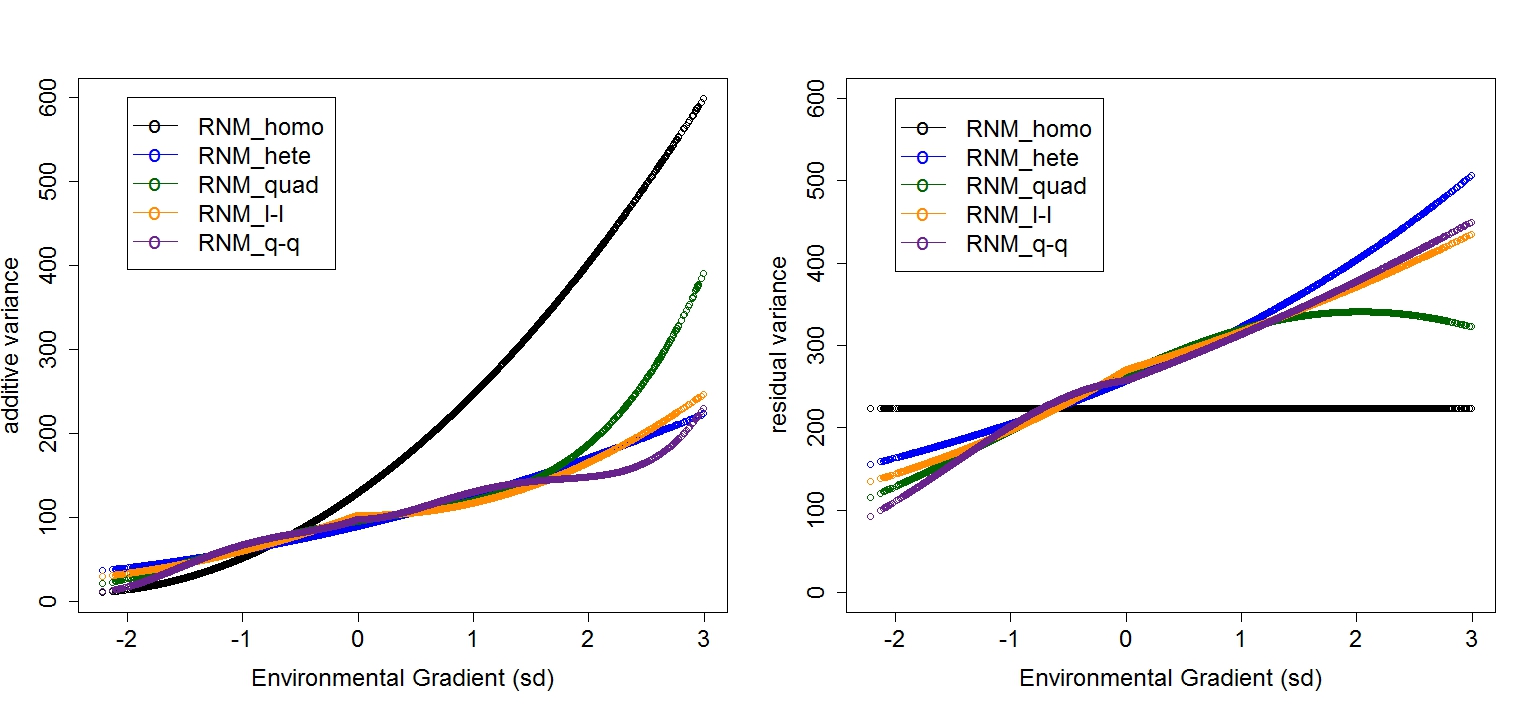

Supplement: Supplementary file 7 — Additional file 7: Figure S6. Additive (left) and residual (right) variance component estimates for post-weaning weight gain of Nellore cattle according to the environmental gradient, for different reaction norm models. RNM_homo: linear homoscedastic; RNM_hete: linear heteroscedastic; RNM_quad: quadratic heteroscedastic; RNM_l-l: spline linear–linear heteroscedastic; RNM_q-q: spline quadratic–quadratic heteroscedastic. [file 12711_2019_470_MOESM7_ESM.jpeg]

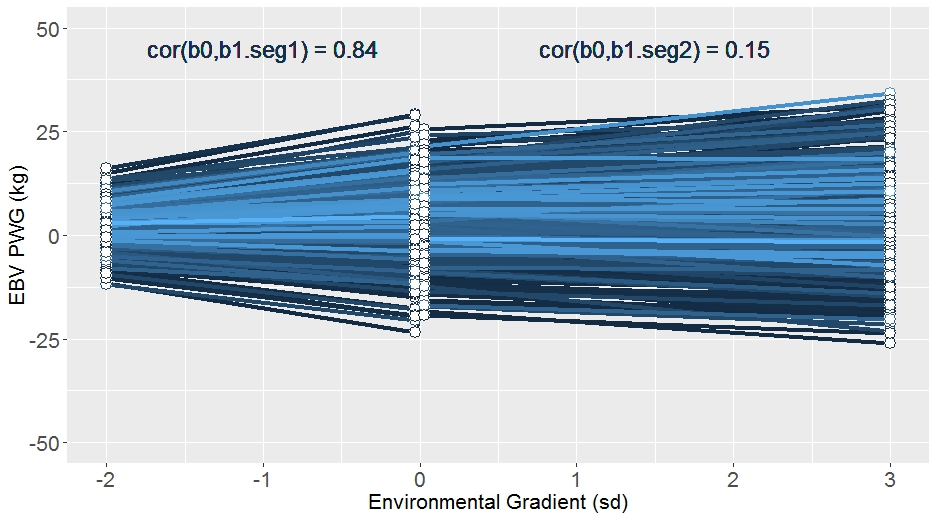

Supplement: Supplementary file 8 — Additional file 8: Figure S7. Estimated breeding values (EBV) for post-weaning weight gain (PWG) of Nellore cattle according to the environmental gradient (EG), obtained with a heteroscedastic linear reaction norm model (RNM_hete). To generate the results of this plot, data of the two segments (EG < 0 and EG > 0) were analyzed separately, running two independent RNM_hete analyses. The correlation estimates between the intercept (b0) and slope of the two segments (b1.seg1 and b1.seg2) are provided. Reaction norms of genotyped sires with at least 50 progeny and in common among the two EG segments (n = 621) are presented. [file 12711_2019_470_MOESM8_ESM.jpeg]

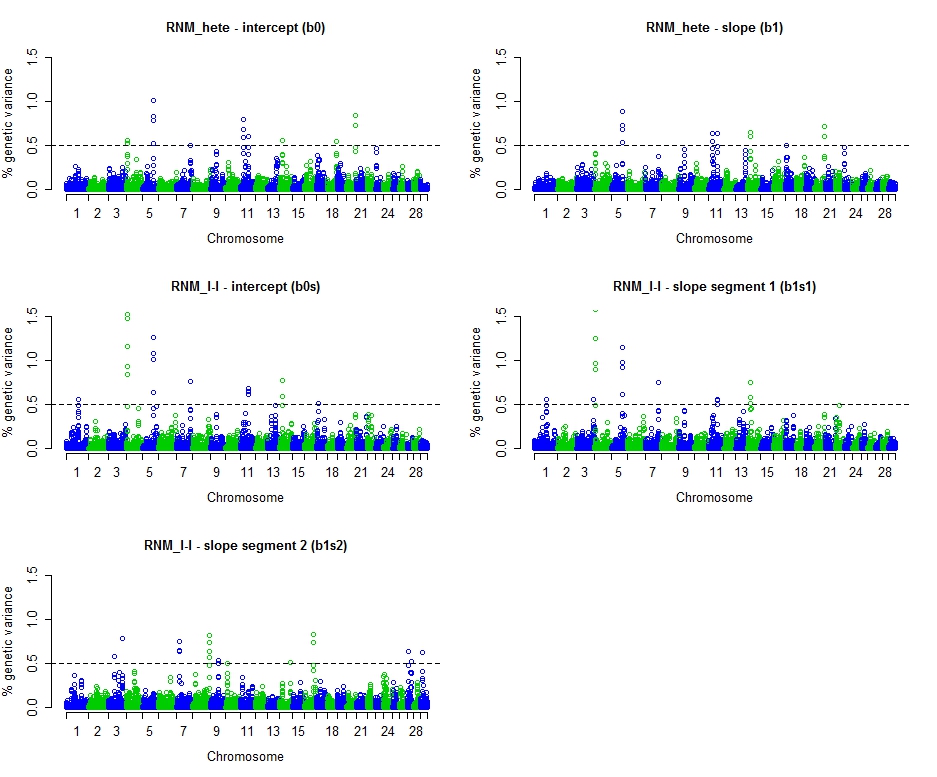

Supplement: Supplementary file 9 — Additional file 9: Figure S8. Manhattan plots of percentage of genetic variance explained by the segment of 5 adjacent SNPs, for each parameter of the reaction norm models RNM_hete and RNM_l-l (RNM_hete: heteroscedastic linear; RNM_l-l: heteroscedastic spline linear–linear). The horizontal dashed line at 0.5 represents the empirical threshold used to identify candidate genomic regions (≥ 0.5). [file 12711_2019_470_MOESM9_ESM.jpeg]

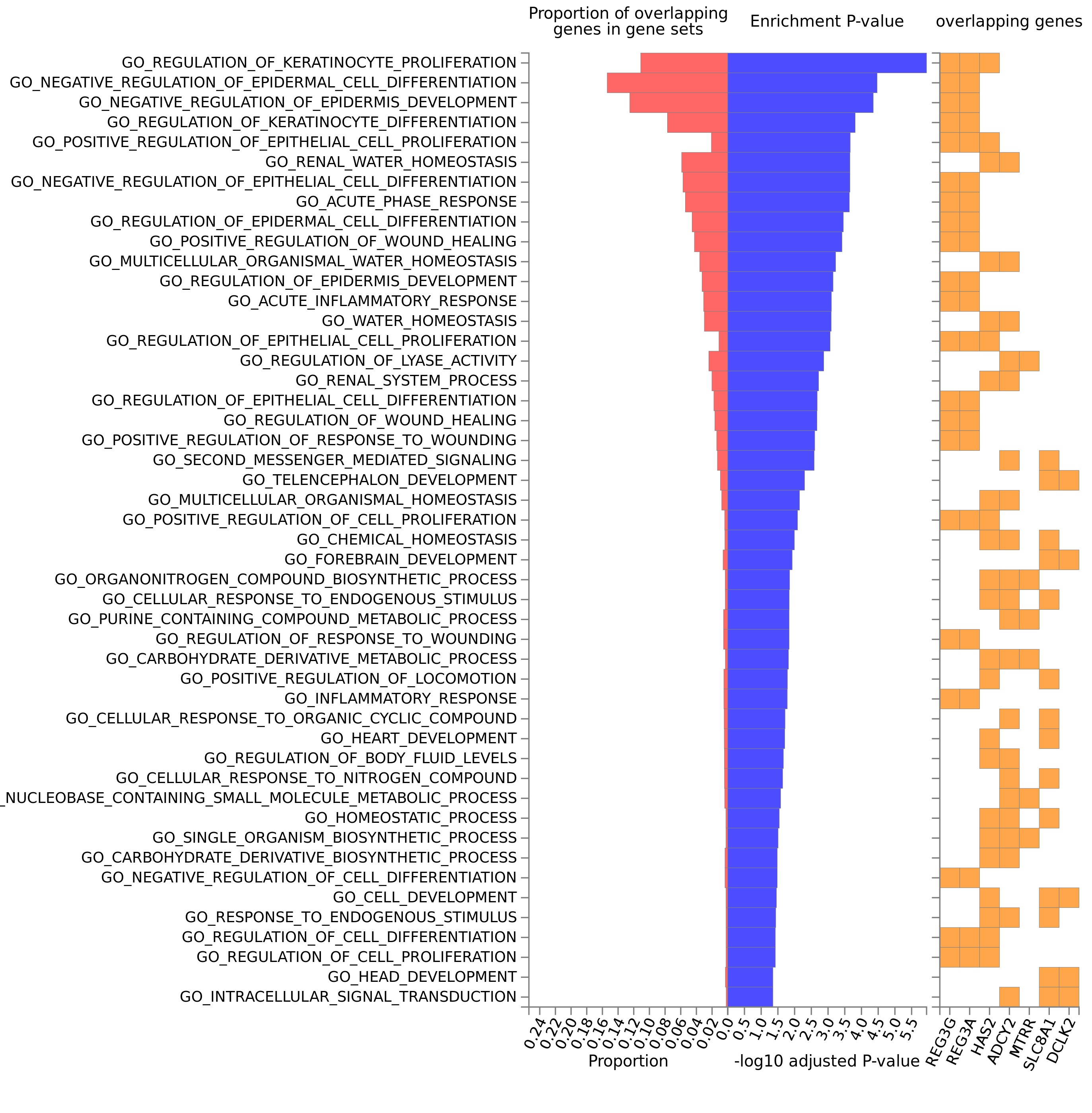

Supplement: Supplementary file 10 — Additional file 10: Figure S9. Significantly enriched gene ontology biological processes, identified by GENE2FUNC process of FUMA, associated with candidate genes for sensitivity to environmental variation of post-weaning weight gain in Nellore cattle, assessed by a random slope (b1) of a heteroscedastic linear random regression model (RNM_hete). [file 12711_2019_470_MOESM10_ESM.jpeg]

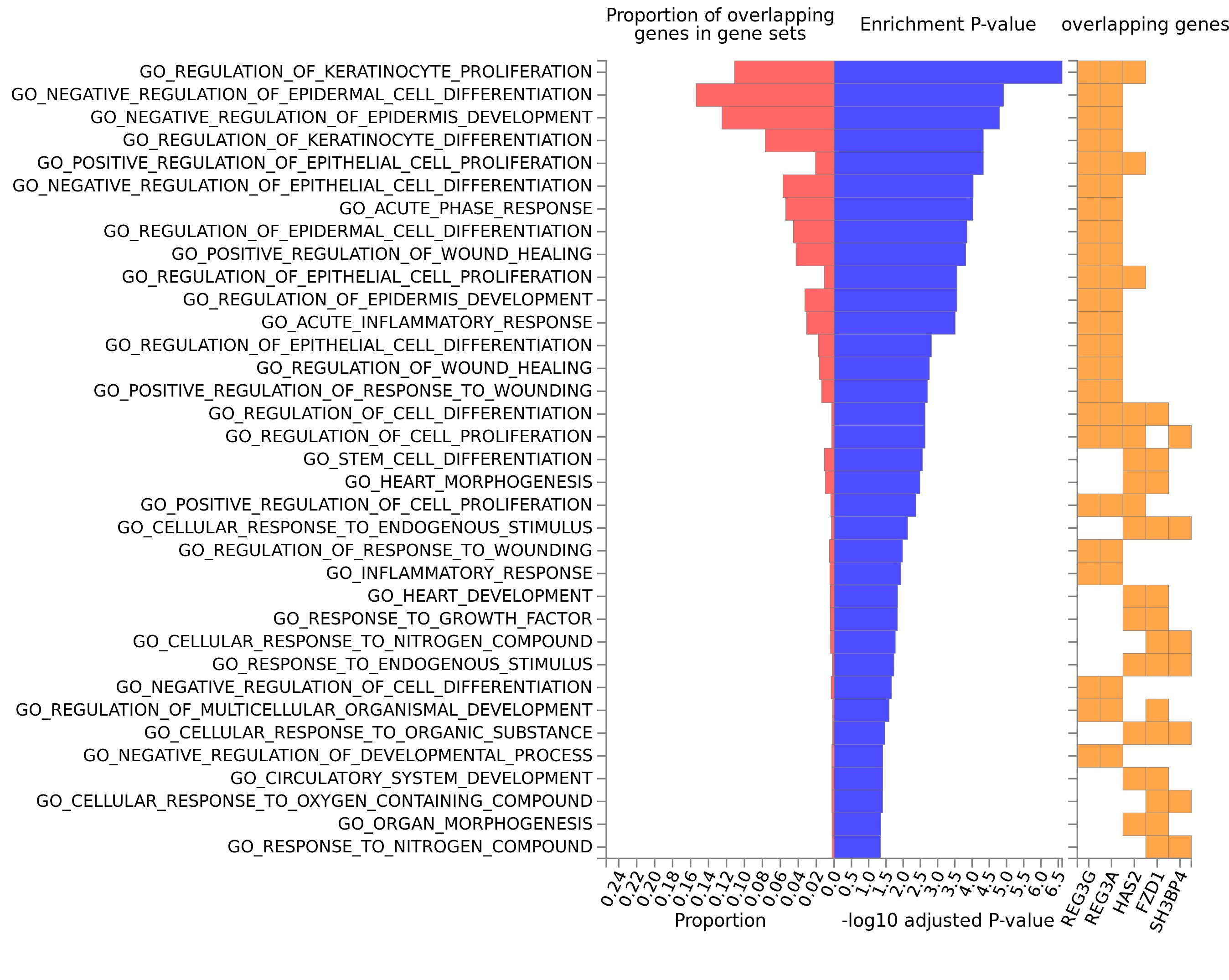

Supplement: Supplementary file 11 — Additional file 11: Figure S10. Significantly enriched gene ontology biological processes, identified by GENE2FUNC process of FUMA, associated with candidate genes for sensitivity to environmental variation of post-weaning weight gain in Nellore cattle, assessed by a random slope of the first segment (b1seg1) of a heteroscedastic spline linear–linear random regression model (RNM_l-l). [file 12711_2019_470_MOESM11_ESM.jpeg]

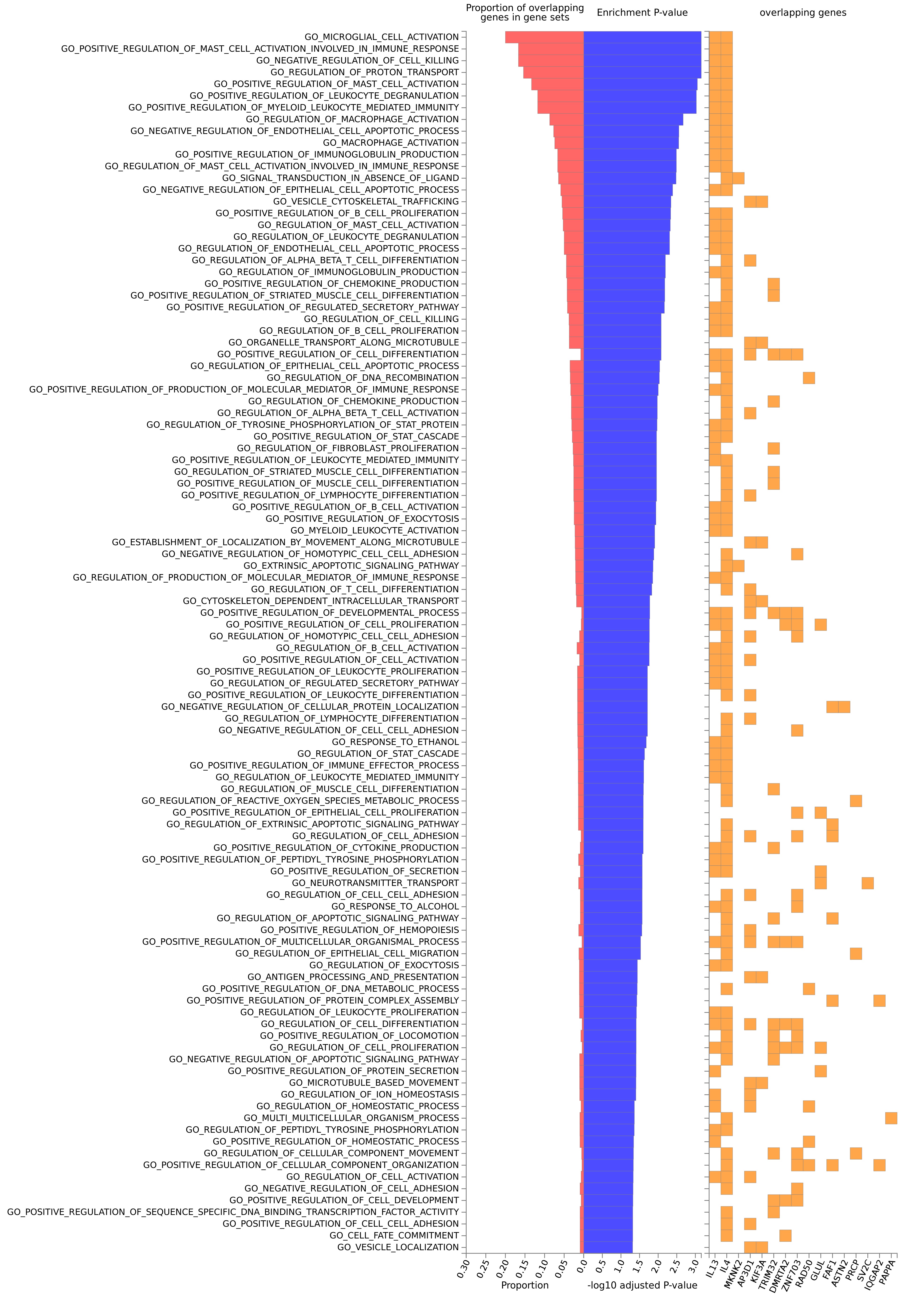

Supplement: Supplementary file 12 — Additional file 12: Figure S11. Significantly enriched gene ontology biological processes, identified by GENE2FUNC process of FUMA, associated with candidate genes for sensitivity to environmental variation of post-weaning weight gain in Nellore cattle, assessed by a random slope of the second segment (b1seg2) of a heteroscedastic spline linear–linear random regression model (RNM_l-l). [file 12711_2019_470_MOESM12_ESM.jpeg]

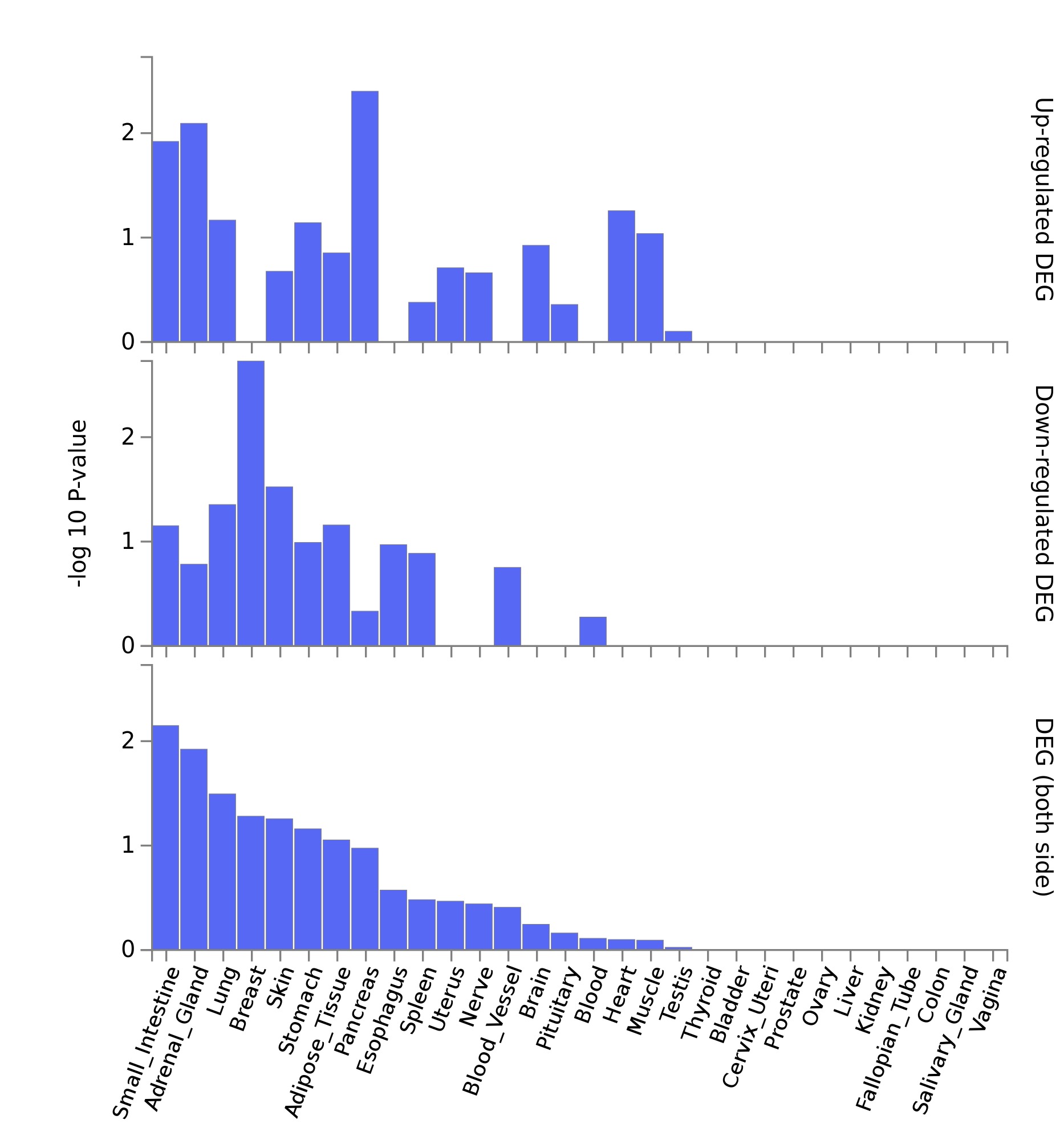

Supplement: Supplementary file 13 — Additional file 13: Figure S12. Enrichment of human tissue specific gene expression based on GTEx v6 RNA-seq data [36], obtained with GENE2FUNC process of FUMA, for candidate ortholog bovine genes associated with sensitivity to environmental variation of post-weaning weight gain in Nellore cattle, assessed by a random slope (b1) of a heteroscedastic linear random regression model (RNM_hete). [file 12711_2019_470_MOESM13_ESM.jpeg]

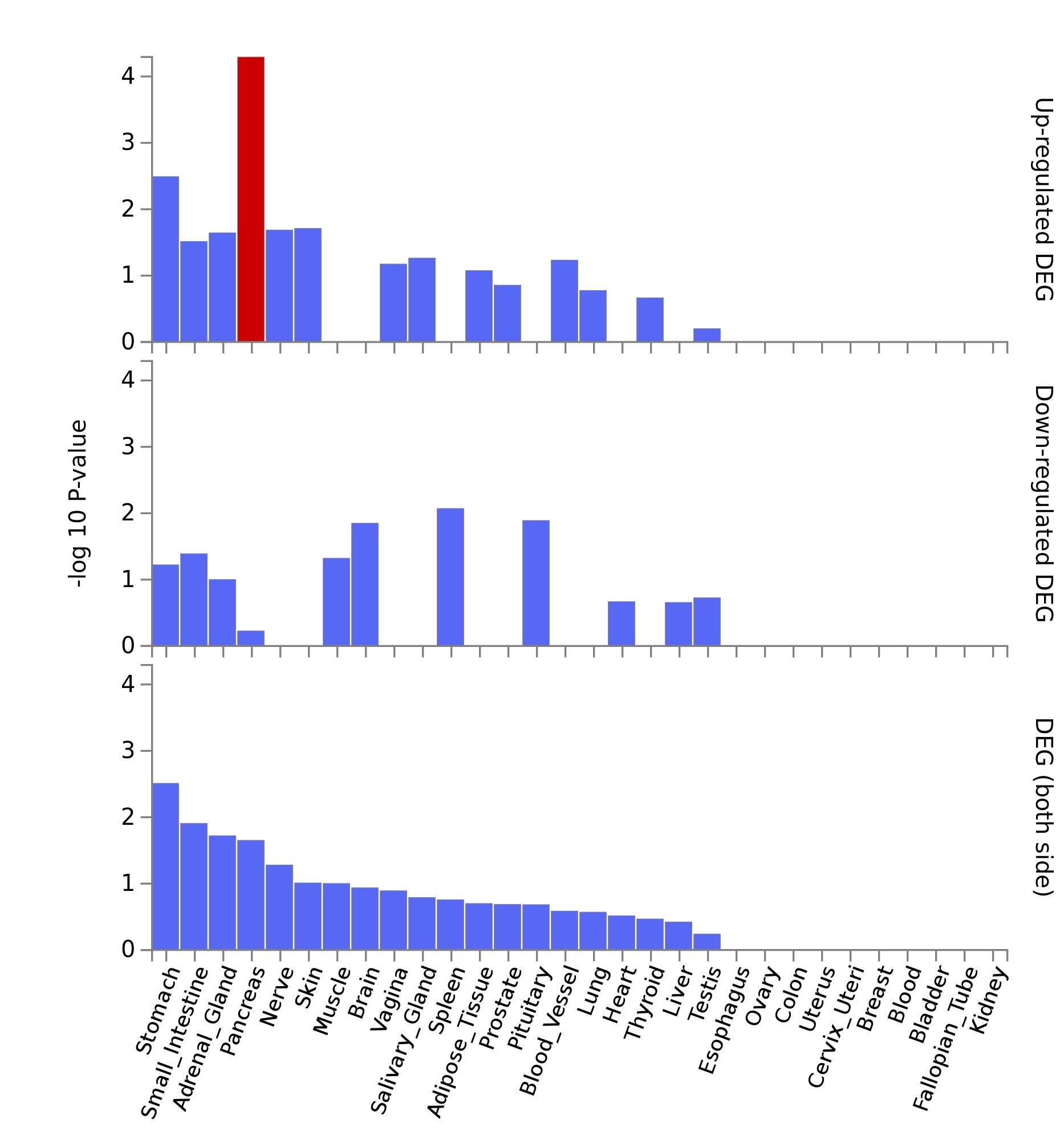

Supplement: Supplementary file 14 — Additional file 14: Figure S13. Enrichment of human tissue specific gene expression based on GTEx v6 RNA-seq data [36], obtained with GENE2FUNC process of FUMA, for candidate ortholog bovine genes associated with sensitivity to environmental variation of post-weaning weight gain in Nellore cattle, assessed by a random slope of the first segment (b1seg1) of a heteroscedastic spline linear–linear random regression model (RNM_l-l). Significant enrichment at Bonferroni corrected P ≤ 0.05 are colored in red. [file 12711_2019_470_MOESM14_ESM.jpeg]

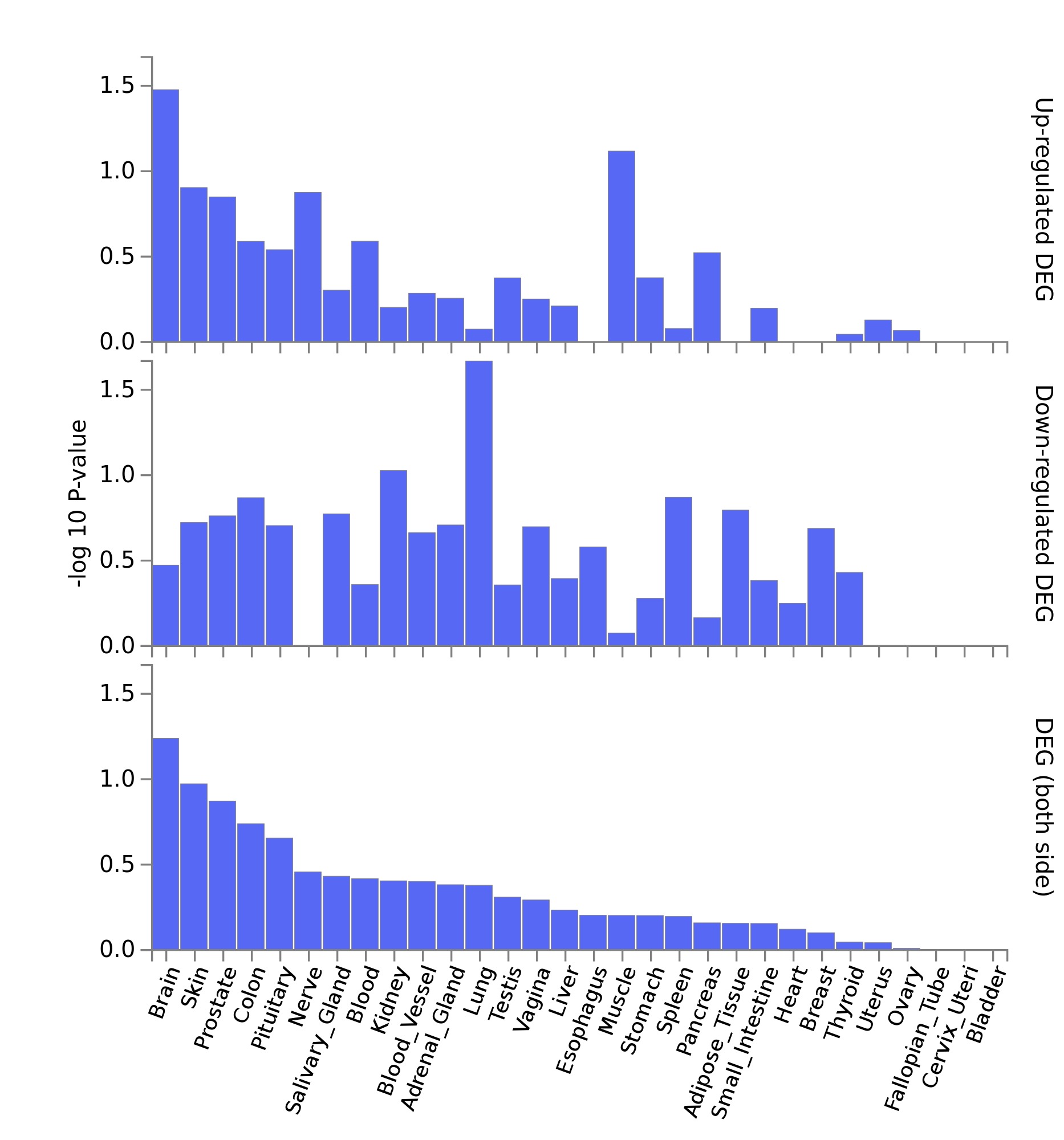

Supplement: Supplementary file 15 — Additional file 15: Figure S14. Enrichment of human tissue specific gene expression based on GTEx v6 RNA-seq data [36], obtained with GENE2FUNC process of FUMA, for candidate ortholog bovine genes associated with sensitivity to environmental variation of post-weaning weight gain in Nellore cattle, assessed by a random slope of the second segment (b1seg2) of a heteroscedastic spline linear–linear random regression model (RNM_l-l). [file 12711_2019_470_MOESM15_ESM.jpeg]

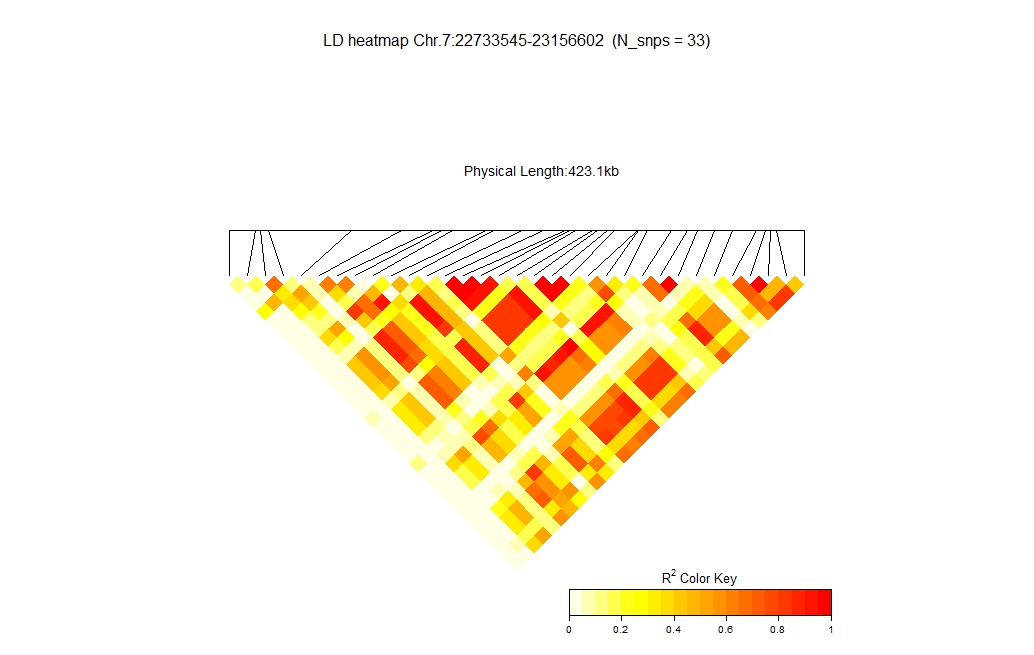

Supplement: Supplementary file 16 — Additional file 16: Figure S15. Linkage disequilibrium (r2) between markers of the 22.7-23.2 Mb region of chromosome 7 (UMD 3.1 assembly), in Nellore cattle. [file 12711_2019_470_MOESM16_ESM.jpeg]
